# Structural and immunologic correlates of chemically stabilized HIV-1 envelope glycoproteins

**DOI:** 10.1371/journal.ppat.1006986

**Published:** 2018-05-10

**Authors:** Torben Schiffner, Jesper Pallesen, Rebecca A. Russell, Jonathan Dodd, Natalia de Val, Celia C. LaBranche, David Montefiori, Georgia D. Tomaras, Xiaoying Shen, Scarlett L. Harris, Amin E. Moghaddam, Oleksandr Kalyuzhniy, Rogier W. Sanders, Laura E. McCoy, John P. Moore, Andrew B. Ward, Quentin J. Sattentau

**Affiliations:** 1 The Sir William Dunn School of Pathology, The University of Oxford, Oxford, United Kingdom; 2 Department of Integrative Structural and Computational Biology, IAVI Neutralizing Antibody Center, Collaboration for AIDS Vaccine Discovery and Center for HIV/AIDS Vaccine Immunology and Immunogen Discovery, The Scripps Research Institute, La Jolla, California, United States of America; 3 Center for Molecular Microscopy (CMM), National Cancer Institute (NCI), Leidos Biomedical Research, Inc., Frederick National Laboratory for Cancer Research, Frederick, Maryland, United States of America; 4 Department of Surgery, Duke University Medical Center, Durham, North Carolina, United States of America; 5 Duke Human Vaccine Institute, Department of Medicine, Duke University School of Medicine, Durham, North Carolina, United States of America; 6 Departments of Immunology and Molecular Genetics and Microbiology, Duke University Medical Center, Durham, North Carolina, United States of America; 7 Center for HIV/AIDS Vaccine Immunology and Immunogen Discovery (CHAVI-ID), The Scripps Research Institute, La Jolla, California, United States of America; 8 IAVI Neutralizing Antibody Center and the Collaboration for AIDS Vaccine Discovery (CAVD), The Scripps Research Institute, La Jolla, California, United States of America; 9 Department of Medical Microbiology, Academic Medical Center, University of Amsterdam, Amsterdam, The Netherlands; 10 Division of Infection and Immunity, University College London, London, United Kingdom; 11 Department of Microbiology and Immunology, Weill Medical College of Cornell University, New York, New York, United States of America; Miller School of Medicine, UNITED STATES

## Abstract

Inducing broad spectrum neutralizing antibodies against challenging pathogens such as HIV-1 is a major vaccine design goal, but may be hindered by conformational instability within viral envelope glycoproteins (Env). Chemical cross-linking is widely used for vaccine antigen stabilization, but how this process affects structure, antigenicity and immunogenicity is poorly understood and its use remains entirely empirical. We have solved the first cryo-EM structure of a cross-linked vaccine antigen. The 4.2 Å structure of HIV-1 BG505 SOSIP soluble recombinant Env in complex with a CD4 binding site-specific broadly neutralizing antibody (bNAb) Fab fragment reveals how cross-linking affects key properties of the trimer. We observed density corresponding to highly specific glutaraldehyde (GLA) cross-links between gp120 monomers at the trimer apex and between gp120 and gp41 at the trimer interface that had strikingly little impact on overall trimer conformation, but critically enhanced trimer stability and improved Env antigenicity. Cross-links were also observed within gp120 at sites associated with the N241/N289 glycan hole that locally modified trimer antigenicity. In immunogenicity studies, the neutralizing antibody response to cross-linked trimers showed modest but significantly greater breadth against a global panel of difficult-to-neutralize Tier-2 heterologous viruses. Moreover, the specificity of autologous Tier-2 neutralization was modified away from the N241/N289 glycan hole, implying a novel specificity. Finally, we have investigated for the first time T helper cell responses to next-generation soluble trimers, and report on vaccine-relevant immunodominant responses to epitopes within BG505 that are modified by cross-linking. Elucidation of the structural correlates of a cross-linked viral glycoprotein will allow more rational use of this methodology for vaccine design, and reveals a strategy with promise for eliciting neutralizing antibodies needed for an effective HIV-1 vaccine.

## Author summary

Chemical cross-linking has been used for almost a century for vaccine preparation, and is still common today for inactivation and stabilization of vaccine antigens. Despite this, cross-linking is used empirically and very little is understood of its effects on antigen structure and how this may modify immune responses. The HIV-1 envelope glycoproteins are the only antigens that can elicit neutralizing antibodies, and are therefore a major focus of vaccine design. Here we have chemically cross-linked the envelope glycoprotein trimer of HIV-1, and observe using high-resolution electron microscopy that cross-links are formed at very specific sites within the trimer, stabilizing but preserving its overall conformation. Trimer stabilization resulted in increased neutralization breadth of difficult to neutralize heterologous viruses, which is the major goal of antibody-based vaccine design. They also affected helper T cell epitope immunodominance, an observation of relevance to optimizing vaccine design to elicit T cell help for B cell responses. These results reveal for the first time the structural effects of cross-linking on a highly conformational vaccine antigen and pave the way for more rational use of this technology in vaccine design for HIV-1 and other pathogens.

## Introduction

Chemical cross-linking has been used for almost a century for inactivation, detoxification and stabilization of vaccine antigens [1], and remains in wide use for inactivated polio [2] and influenza [3] viruses and diphtheria (DT), pertussis (PT) and tetanus toxins (TT) [4]. Cross-linking technology continues to be used empirically, with endpoints defined as infectivity reduction for inactivated pathogens, or depletion of enzyme activity for toxins, with largely unknown effects on antigen structure, antigenicity and immunogenicity by comparison with the non-cross-linked counterpart. Although some vaccine specifications may include antigenic conservation such as for hemagglutinin (HA) in inactivated influenza vaccines [5, 6], little is otherwise known of the structural and immunologic impacts of cross-linking on vaccine antigens. Acquisition of this knowledge is important, as it would allow more rational translation of chemical cross-linking approaches to the design and development of future vaccines.

HIV-1 is the cause of AIDS and is responsible for a global pandemic estimated in 2015 at 37 million infected people. A prophylactic vaccine would be a cost-effective means to reduce or eliminate the pandemic, but its development has been challenging [7, 8]. Infusion of neutralizing antibodies (NAb) mediates robust protection against immunodeficiency virus infection in animal models [9, 10], providing proof-of-concept for adopting active NAb induction as a prophylactic vaccination strategy. The only HIV-1 NAb target is the envelope glycoprotein (Env), a non-covalently linked trimer of gp120 (surface) and gp41 (transmembrane) glycoprotein heterodimers. Soluble recombinant Env trimers have been designed for vaccine use that are close structural and antigenic mimics of native, membrane-anchored Env, as exemplified by the prototype SOSIP.664 trimer derived from the BG505 viral clone [11–15]. The soluble BG505 SOSIP Env trimer presents all known conserved broadly neutralizing antibody (bNAb) epitope clusters that are present in the truncated sequence, but intrinsically masks most non-neutralizing (non-NAb) epitopes [11]. Immunization of rabbits and macaques with BG505 SOSIP trimer induced for the first time NAbs against autologous, difficult-to-neutralize (Tier-2) BG505 pseudovirus (PV) [16–19]. Tier-2 PV represent clinically relevant circulating viral strains whose neutralization is a major goal in HIV-1 vaccine design [20].

Although relatively thermodynamically stable in solution, BG505 SOSIP trimers sample different conformations that may reduce B cell recognition of neutralizing antibody epitopes via an immune evasion strategy termed conformational masking [17, 21–23]. Moreover, trimer stability may be influenced by co-formulation with some adjuvants [18, 24], and maintenance of antigenic integrity in vivo is unknown, but likely to be adversely modified over time. A current focus is therefore to prepare stable and homogeneous soluble Env trimers that may drive B cells to elicit bNAbs. Structure-based mutagenesis has yielded Env trimers with improved stability, antigenicity [17, 18, 25, 26] and immunogenicity [17, 26] but these have yet to induce bNAbs. An alternative or complementary approach is stabilization by chemical cross-linking. We showed previously that GLA cross-linking of soluble first-generation Env trimers enhanced stability and reduced exposure of non-NAb epitopes [27]. Chemical cross-linking has subsequently been applied empirically to improve both membrane-anchored [28] and soluble [18, 29] HIV-1 Env stability.

Despite this promise, chemical cross-linking of vaccine antigens lacks underpinning by structural biology and correlates of adaptive immunity. To address this gap in knowledge, we used cryo-electron microscopy (cryo-EM) to generate the first high-resolution 4.2 Å structure of a chemically cross-linked vaccine antigen. GLA cross-linked soluble BG505 SOSIP trimer (from hereon termed GLA-SOSIP trimer) revealed near structural identity with its unmodified counterpart (from hereon termed SOSIP trimer), but containing defined inter- and intra-subunit cross-links that stabilized the structure and improved antigenicity. Immunization of rabbits and mice with GLA-SOSIP trimer elicited antibody and T helper cell (Th) responses that differed in magnitude and specificity from those induced by SOSIP trimer, and showed modest, but significantly broadened heterologous neutralization of difficult to neutralize Tier-2 viruses representative of the global pandemic. Our data reveal the first molecular snapshot of a chemically cross-linked vaccine antigen and the adaptive immune response elicited by it, and suggest a path forward for the use of chemical cross-linking in rational improvement of HIV-1 Env-based and other vaccine antigens.

## Methods

### Antibodies and proteins

Antibodies b12 [30], NIH45-46 [31], VRC01, VRC03 [32], 412D [33], A32 [34], C11 [35], CH01 [36], PGT145 [31], 2G12 [37], PGT121 [31], PGT128 [31], PGT135 [31], 14E, 19b, 39F [38], 35022 [39], 3BC176, 3BC315 [40], PGT151 [41], CAP256-VRC26.08 [42], PDGM1400 [43] and 7B2 [44] were expressed in freestyle 293F cells under serum-free conditions and purified by protein A chromatography as previously described [45]. Rabbit mAbs 10A, 11A and 11B were isolated and prepared as described [46]. Soluble CD4 (sCD4) [47], CD4-IgG2 [48], 15e, F105, 17b [49], PG16 [50] and b6 [30] were from the IAVI Neutralizing Antibody Consortium. Antibodies HGN194, HR10 and HJ16 [51] were a kind gift from D. Corti and A. Lanzavecchia. The following fragment antibody-binding (Fabs) for SPR were produced by expression in Freestyle 293F cells and were a kind gift of W. Schief: 39F [38], 4025 [52], PGT121 [53], PGT128 [53], PGT145 [31], PG16 [50], PGT151 [41], 3BC315 [40], 35022 [39], 8ANC195 [31], VRC01 [32], PGV04 [54], NIH45-46 [31], 3BNC60 [31], B6 [55]. Antibodies were biotinylated using EZ-link NHS-LC-Biotin according to the manufacturer’s instructions (Fisher Scientific), or were attached to cyanogen bromide-activated agarose using the manufacturer’s protocol (GE Healthcare). BG505 SOSIP.664 gp140 (SOSIP trimer) was expressed in stably transduced CHO or 293T cells and purified as previously described, except that buffers devoid of primary amines were used as previously described [29]. Briefly, proteins were bound to a PGT145 or 2G12 column, eluted with 3 M MgCl_2_ and immediately buffer-exchanged twice into 20 mM HEPES supplemented with 150 mM NaCl, followed by one buffer-exchange into phosphate buffered saline (PBS, Lonza). The eluted trimers were concentrated and purified by size exclusion chromatography (SEC) on a Superdex 200 26/600 or 16/600 column (GE Healthcare) using PBS as the elution buffer. Trimer-containing fractions were pooled and concentrated, passed down a protein A agarose column (Pierce) to remove any potential contaminant human IgG eluted from the column, and flash-frozen in liquid nitrogen and stored at -80°C until use.

### Antigen cross-linking and antibody selection

Unless otherwise specified, GLA cross-linking was performed as previously described [27]. Briefly, SOSIP trimers at 1 mg/mL in PBS were mixed with an equal volume of 15 mM GLA to yield a final concentration of 7.5 mM. After 5 min, 1 M Tris buffer pH 7.4 was added, such that the final concentration was 75 mM. After 10 min, the protein was buffer exchanged into Tris-buffered saline (TBS). The success of the cross-linking procedure was confirmed by reducing SDS polyacrylamide gel electrophoresis (SDS-PAGE) analysis using the NuPAGE system according to the manufacturer’s (Life Technologies) instructions, and as previously described [27]. For immuno-affinity purification, cross-linked proteins were incubated with immobilized PGT151, washed with TBS and bound protein was eluted with 3M MgCl2 and buffer-exchanged into TBS. Following an optional SEC step used for preparation of the material for cryo-EM and immunization, proteins were incubated with immobilized V3 antibodies 19b and/or 14E on columns overnight at 4°C, flow-through was collected, sterile-filtered with Costar Spin-X 0.22 μm filters and stored at 4°C (short-term) or flash-frozen on liquid nitrogen and stored at -80°C. Proteins were analyzed by reducing SDS-PAGE as previously described [27].

### Amine assay

SOSIP or GLA-SOSIP trimer (5 μg) in 20 μL PBS was added to 30 μL of 0.1 M NaHCO_3,_ pH 8.5. 25 μL of 5% 2,4,6-Trinitrobenzene Sulfonic Acid (TNBSA) diluted 1/500 in 0.1 M NaHCO_3_ pH 8.5 was added to the samples for 2 h at 37°C, followed by 25 μL of 10% SDS and 12.5 μL of 1M HCl. Samples were vortexed and the optical density read at 335 nm. The relative quantity of free amines was calculated as (OD_335_ (GLA-SOSIP trimer)–OD_335_ (blank)) / (OD_335_ (SOSIP trimer)–OD_335_ (blank)).

### Cryo-electron microscopy sample preparation

GLA-SOSIP trimers were incubated with a 10-Molar excess of the CD4bs bNAb Fab PGV04 [54] for 1 h at RT. This complex was purified by size exclusion chromatography using a Superose 6 10/30 column (GE Healthcare) in 50 mM Tris, 150 mM NaCl, pH 7.4 buffer. The fractions containing the complex were pooled and concentrated to ~ 2 mg/mL using a 100-kDa cutoff concentrator (Amicon Ultra, Millipore). 1 μL of a *n*-Dodecyl β-D-maltoside (DDM) solution at 1.8 mM was added to 5 μL of this complex, to avoid protein aggregation and to obtain an optimal ice layer on the grid. At 4°C, 3 μL of this mixture (protein + DDM) was applied to a C-Flat 2/2 grid (Electron Microscopy Sciences, Protochips, Inc.), blotted and plunged into liquid ethane using a manual freeze plunger.

### Cryo-electron microscopy data collection

Micrographs were collected on an FEI Titan Krios operating at 300 KeV coupled with Gatan K2 direct electron detector via the Leginon interface [56]. Each exposure image was collected in counting mode at 29000 x nominal magnification resulting in a pixel size of 1.02 Å/pixel, using a dose rate of ~10 e-/pix/sec, and 200 ms exposure per frame. A total of 1329 micrographs were collected in ~72 h. The total dose received for each movie micrograph was 67 e-/Å^2^. The nominal defocus range used was -1.5 to -4.0 μm.

### Cryo-electron microscopy data processing

Movie micrograph frames were aligned using MotionCorr [57] and CTF models were calculated using CTFFIND3 [58]. The resulting motion-corrected and signal-integrated micrographs were subjected to automated difference-of-Gaussian particle selection [59]. The resulting set of molecular projection-image candidates were binned by a factor of 4 and subjected to reference-free class averaging in Relion 1.4b1 [60]. Projection images belonging to structural classes were selected for angular refinement and reconstruction using a low-pass filtered unliganded HIV-1 Env trimer density map as a reference [61]. The projection images were then subjected to 3D classification and split by K-means clustering into six classes. Two classes resulted in stoichiometrically identical maps (Env trimer with three PGV04 Fabs bound) and projection images sorted into these two classes were combined followed by refinement and reconstruction. This data pool was then subjected to particle polishing and the resulting aligned, B-factor-corrected and signal-integrated projection images were refined and reconstructed to a final average resolution of ~4.2 Å (0.143 FSC cut-off).

### Model building

An initial model was generated based on PDB ID 5CEZ [62] and PDB ID 3SE9 [54] using UCSF Chimera [63], Modeler [64] and Coot [65]. A library of 200 homologous 7-mer peptide fragment coordinates per relevant residue were compiled and used in iterative centroid-representation density-guided rebuild-and-refinement in the Rosetta software suite [66]. Rosetta models were manually adjusted in Coot followed by evaluation based on geometry (MolProbity [67]) and cryo-EM density fit (EMRinger [68]). Upon convergence, the best model underwent iterative all-atom Rosetta refinement and manual rebuilding constrained by the cryo-EM density map and with distance constraints introduced for observed cross-links. The final model was selected based on MolProbity, EMRinger, Privateer [69] and CARP [70] validation (S2 Table).

### RMSD analysis

For gp41, gp120 and gp41-gp120 protomers, RMSDs were calculated as mean Cα RMSD between our deposited structure and PDB IDs 4ZMJ, 5CJX, 5ACO, 4TVP, 5I8H, 5CEZ, 5D9Q, 5V7J, 5FYL, 5T3Z, 5UTY, 5T3S, 5V8M and 5V8L. For trimers, RMSDs were calculated as mean Cα RMSD between our deposited structure and PDB IDs 5ACO, 5CEZ, 5V7J, 5FYL, 5T3Z, 5UTY, 5V8M and 5V8L. Secondary structure matching was utilized (“superpose” implementation in Coot run from the python scripting interface).

### Rabbit immunizations

New Zealand White rabbits (*n* = 5 per group) were immunized intramuscularly at Covance Inc. as follows. SOSIP or GLA-SOSIP trimers processed as described above were formulated with Iscomatrix at 75 U/dose in a total volume of 500 μL per dose. Priming and boosting were with 30 μg SOSIP or GLA-SOSIP trimer/dose. Blood samples were collected prior to priming and periodically after each immunization, serum separated, aliquotted and stored at -20°C.

### Mouse immunizations

All experiments used 8–12 week old female BALB/c mice (Charles River or bred at the William Dunn School of Pathology) under specific pathogen-free conditions. Five mice per group were immunized by subcutaneous administration with 10 μg SOSIP- or GLA-SOSIP trimer or vehicle control in a 100 μL PBS/Iscomatrix (0.5 U/dose) formulation on week 0 and 4. Mice were monitored for adverse symptoms throughout. Blood was collected by tail bleed on week 0, 4 and 8, serum separated and stored at -20°C.

### Ethics statement

Animal research using rabbits and mice was carried out in full accordance with local and national ethical guidelines. All protocols for breeding and procedures with mice were approved by the Home Office UK, under the Animals (Scientific Procedures) Act 1986 and Home Office license PPL3003421. Rabbit studies were carried out at Covance Inc.

### Capture ELISA for rabbit sera and human and rabbit mAbs

ELISA plates (Greiner Bio-One) were coated with 4 μg/mL of capture mAb 2G12 at 4°C overnight in PBS. After blocking with 2% BSA/PBS + 0.05% Tween, SOSIP or GLA-SOSIP trimer (0.2 μg/mL) were captured, labelled with a titration series of biotinylated human or rabbit mAbs or sera followed by peroxidase-conjugated detection reagent as appropriate (streptavidin for biotinylated human mAbs or anti-rabbit IgG, Jackson ImmunoResearch). The colorimetric endpoint was obtained using the one-step ultra TMB substrate (Thermo Scientific). MAbs were developed until a signal of approximately 1–2 optical density (OD_450_) units was generated for each antibody, leading to longer incubation periods for non-Nabs, whereas serum endpoint-titer ELISAs were always developed for 10 min. In both cases, color development was stopped with sulfuric acid (0.5 M) and the OD_450_ measured. All ELISA signals were corrected by subtracting the background signal obtained in the absence of primary antibody and the resulting data were plotted against the log_10_ of the antibody concentration using GraphPad Prism V7.0. To generate binding indices from ELISA titration curves, an area under the curve (AUC) analysis of ligand-trimer binding was performed; the binding index represents the ratio of cross-linked trimer value to the value of the matched unmodified SOSIP trimer that was used for cross-linking. Binding indices were calculated as (AUC(GLA-SOSIP trimer)—AUC(blank)) / (AUC(SOSIP trimer)—AUC(blank)), where blank = negative control curve of the respective mAb without antigen. Indices <1 indicate reduced binding to the cross-linked trimer compared to its unmodified counterpart, and the converse for values >1. Induction ELISAs with sCD4 were performed similarly, with 1 μg/mL sCD4 added to half the replicate wells. Induction scores were calculated as (AUC (trimer+sCD4)–AUC (blank+sCD4))—(AUC (trimer-sCD4)–AUC (blank-sCD4)) where AUC (trimer+sCD4) and AUC (trimer-sCD4) are the AUC of mAb binding to trimer-containing wells in presence and absence of sCD4 respectively, and AUC (blank+sCD4) and AUC (blank-sCD4) are the AUC of negative control wells without any trimer in presence and absence of sCD4, respectively. Induction scores were normalized for inter-experiment differences by dividing by the AUC against 2G12 before calculating mean scores of 3 independent repeats. The cross-competition ELISA was performed as above, but rabbit sera were added to wells at 1:30 dilution, immediately followed by biotinylated competition mAb. Maximum percent inhibition values (MPI) were calculated as (1- (OD_450_(serum)–OD_450_(neg)) / (OD_450_(pos)–OD_450_(neg))) x 100%, where OD_450_ = optical density measured at 450 nm, neg = negative control wells without biotinylated mAb and pos = positive control wells without competing serum.

### Surface plasmon resonance (SPR)

Kinetics and affinity of antibody-antigen interactions were measured on a ProteOn XPR36 instrument (Bio-Rad) using GLC Sensor Chip (Bio-Rad) and 1x HBS-EP+ pH 7.4 running buffer (20x stock from Teknova, Cat. No H8022) supplemented with BSA at 1 mg/mL. Chip surfaces were prepared using the Human Antibody Capture Kit according to manufacturer’s instructions (Cat. No BR-1008-39 from GE) to immobilize approximately 6000 response units (RUs) of capture antibody onto all 6 flow cells of the GLC Chip. In each cycle, 2G12 mAb was captured for 120 s at 2 μg/mL and approximately 600 RUs of trimer were captured at 10 μg/mL for 120 s. Dilution series of various Fabs were passed over the surface for 180 s, followed by 600 s of buffer. Four injections of 3 M Magnesium Chloride for 180 s were used to regenerate the surface after each cycle. Raw sensograms were analyzed using ProteOn Manager software (Bio-Rad). Following interspot and column double referencing, kinetics were fitted to a Langmuir 1:1 binding model where applicable. SPR signal ratios were defined as: signal_observed_ / MW_Fab_ / signal_capture_ x MW_SOSIP_ x F_stoich_ where signal_observed_ is the maximum signal observed during Fab injection, signal_capture_ is the signal of the SOSIP capture, MW_Fab_ is the molecular weight of the FAb and MW_SOSIP_ is the molecular weight of one SOSIP protomer and F_stoich_ is a correction factor for mAbs that do not bind in a 1:1 stoichiometry according to published literature (3:1 = 3 for PG16 and PGT145 [71] and 3:2 = 1.5 for PGT151 [72] and 3BC315 [73]). SPR binding indices were calculated as signal ratio (GLA-SOSIP trimer) / signal ratio (SOSIP trimer) where 1 is no change in binding and 0 is complete loss of binding.

### Endpoint ELISA for mouse sera

Detection of mouse WT-specific serum antibodies was performed using endpoint titer ELISA. 2G12 antibody (4 μg/mL, 50 μL/well) was captured overnight at 4°C onto high-protein-binding ELISA plates (Spectraplate 96HB, Perkin Elmer). Plates were washed in PBS/Tween (0.05% v/v) and wells blocked using BSA (2% w/v; 200 μL/well) for 2 h at RT, and washed. SOSIP trimer (0.2 μg/mL, 50 μL/well) was added for 2 h at RT and washed as before. Mouse serum samples diluted in PBS/BSA (1% w/v) starting at 1:100 then stepwise 5-fold were added to the ELISA plates (50 μL/well) and incubated overnight at 4°C. Plates were washed and Peroxidase-conjugated rabbit anti-mouse IgG antibody (1:5000; 50 μL/well, Jackson Immunoresearch) added to all wells for 1 hr at RT. Plates were washed and TMB substrate (50 μL/well, Thermofisher Scientific) added to all wells. Color development was monitored and terminated using sulphuric acid (0.5 M; 50 μL/well). Optical density (OD) values for each well were measured at 450 nm and 570 nm and calculated as OD_450-570nm_. Background values (OD_no serum_) were subtracted from sample readings. Endpoint titers were calculated using non-linear regression curve fitting and interpolated values transformed into log_10_ endpoint titers (Graphpad Prism 7 for Mac).

### TZM-bl neutralization assays and mutant PV

Briefly, PV generated using Tier-1, homologous BG505 Tier-2, or global panel Tier-2 *envs* [74] were reacted with titrations of week 51 sera and neutralization activity determined using the TZM-bl reporter assay [75]. Luciferase expression was measured after 2 days and IC_50_ values were determined as the serum concentration that reduced the background-subtracted relative light units (RLU) by 50% compared to virus-only control wells. PV expressing Env with mutations S241N, P291T and S241N + P291T were used for mapping autologous neutralization specificity as described above.

### Linear peptide microarray mapping and data analysis

Solid phase peptide microarray epitope mapping was performed as previously described [76, 77] with minor modifications. Briefly, array slides were prepared by JPT Peptide Technologies GmbH (Germany) by printing a library designed by Dr. B. Korber, Los Alamos National Laboratory, onto Epoxy glass slides (PolyAn GmbH, Germany). The library contains 15-mer peptides overlapping by 12, covering consensus Env (gp160) clade A, B, C, D, Group M, CRF1, and CRF2 and vaccine strains (gp120) 1.A244, 1.TH023, MN, C.1086, C.TV1, and C.ZM651. Sera were diluted 1/50 and applied to the peptide array, followed by washing and detection using goat anti-human IgG-Alexa Fluor 647. Array slides were scanned at a wavelength of 635 nm with an InnoScan 710 AL scanner (Innopsys, France) using XDR mode. Scan images were analyzed using MagPix 8.0 software to obtain binding intensity values for all peptides. Binding of post-immunization serum to each peptide was subtracted from its baseline value, which was defined as the median signal intensity of the triplicates of the peptide for the matched pre-bleed serum + 3 x standard error of the triplicates. Binding magnitude to each identified epitope was defined as the highest binding by a single peptide within the epitope region.

### T cell proliferation and peptide mapping analysis

Spleens were harvested from mice 4 weeks after the boost, dissected using aseptic technique, and single cells isolated by passing through a 100 μm filter. CD4 T cells were isolated by negative selection using magnetic beads following the manufacturer’s instructions (Miltenyi Biotech). CD4 T cell-depleted splenocyte populations were γ-irradiated (3000 rads) and used as antigen-presenting cells (APCs). Peptide specificity of SOSIP trimer-specific CD4 T cells was determined using a 165-peptide library spanning the entire BG505 SOSIP Env amino acid sequence (Chempeptide Inc.). Each peptide comprised 15-amino acids overlapping with adjacent peptides by 5 residues. CD4 T cells (10^6^ cells/well) and APCs (10^5^ cells/well) were pipetted into 96-well plates with medium only, SOSIP trimer (10 μg/mL), GLA-SOSIP trimer (10 μg/mL), or individual peptides (10 μg/mL) and incubated for 4 days. Supernatants were harvested and stored at -80°C prior to assay for cytokines by ELISA. ^3^H-thymidine (1 μCi, 20 μL/well) was then added to all wells and plates were incubated for a further 24 h prior to harvesting. Cells were harvested onto filter mats using a Micro96 harvester (Skatron Instruments) and radioactivity detected on a 1450 LSC Microbeta Trilux (Perkin Elmer). Counts per minute (CPM) for each well were calculated as CPM_test_—CPM_no antigen_.

### Cytokine sandwich ELISA

IFN-γ and IL-4 were detected in restimulated CD4 T cell supernatants using specific sandwich ELISAs following the manufacturer’s instructions (Thermofisher Scientific). OD_450-570nm_ values for each well were calculated as OD_test_—OD_no antigen_, and were converted into concentrations using standard curves.

### Statistical analyses

Statistical analysis was performed in Prism using the tests described in the corresponding figure legends. Briefly, one-way ANOVA of log-transformed data with Sidak’s post-test correction to account for multiple comparisons were used to analyze normally distributed data including cross-competition scores and log-transformed endpoint titers. Non-parametric analysis (not assuming a Gaussian distribution) between two independent groups was performed using a two-tailed Mann-Whitney U test and an unmatched, unpaired Kruskal-Wallis test with Dunn’s multiple comparison test was used to compare non-normally distributed data with more than one comparison.

## Results

### Modification and antigenicity of GLA-SOSIP trimer

**Fig 1:**
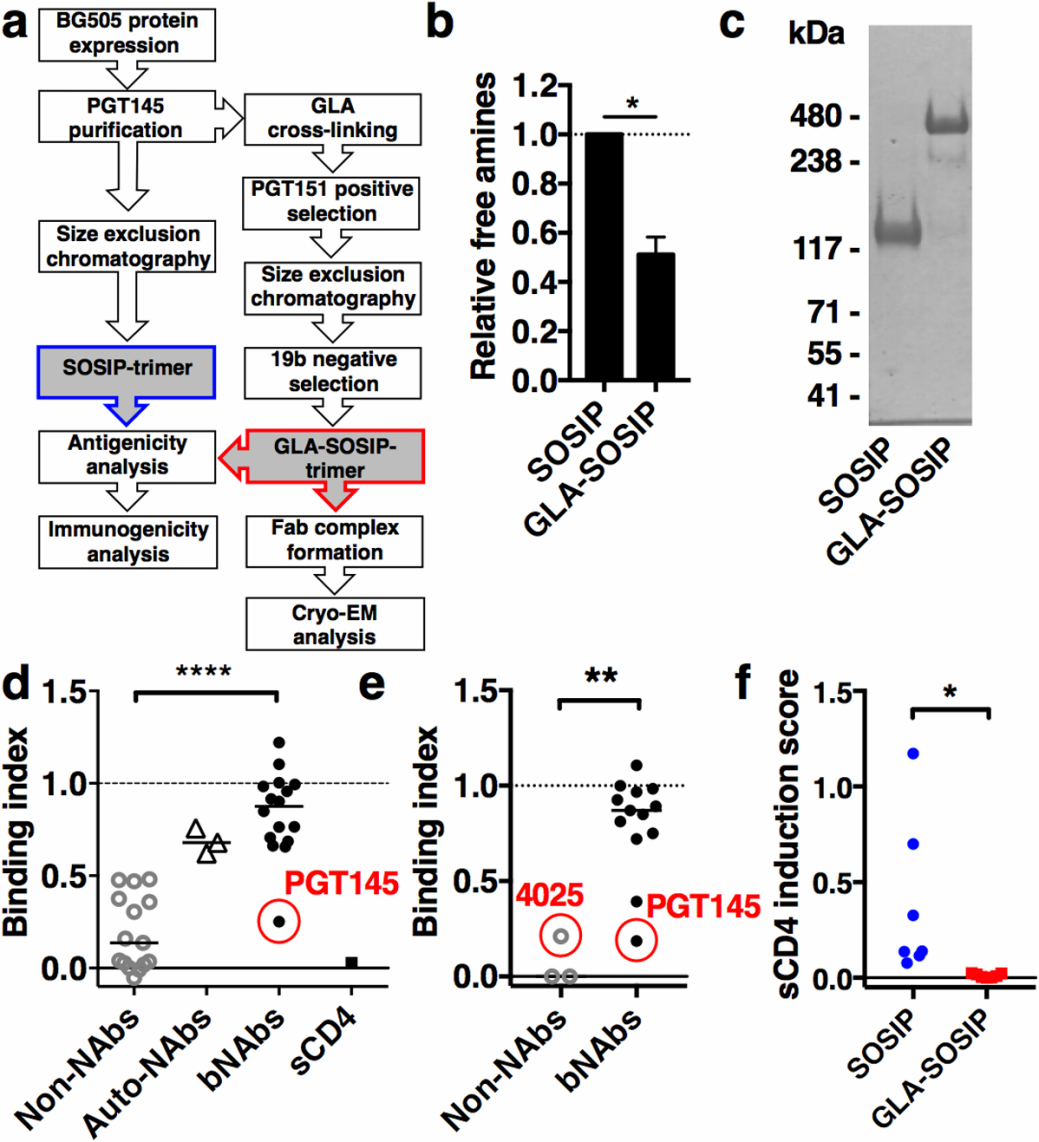
Modification, stability and antigenicity of GLA-SOSIP trimer. (**a**) Strategy for selection and analysis of SOSIP and GLA-SOSIP trimers. (**b**) ELISA-based quantification of free amines in GLA-SOSIP trimers normalized to SOSIP-trimers (SOSIP). *p<0.05 2-tailed *t*-test. (**c**) Reducing SDS-PAGE analysis with molecular weight marker sizes indicated. ELISA (**d**) and SPR (**e**) binding indices calculated as ratios of binding of mAbs to SOSIP trimers / GLA-SOSIP trimer for non-Nabs, autologous rabbit Nabs (Auto-NAbs), bNAbs and sCD4, where 1 = no change of relative binding and 0 = complete loss of binding; ****p<0.0001, **p<0.01, Kruskal-Wallis test with Dunn’s multiple-comparison correction. PGT145 bNAb is highlighted in a red circle in (**d**) and PGT145 and 4025 in (**e**). Horizontal bars represent median, ELISA data combined from 2–4 independent experiments each performed with technical duplicates. Unprocessed ELISA and SPR data are in S1 and S2 Figs, respectively and SPR-derived binding constants and signal ratios in S1 Table. (**f**) sCD4-induced non-Nab binding. Changes in binding of V3-specific and CD4i non-Nabs upon sCD4 induction were measured by ELISA. sCD4 induction scores show the difference between normalized area-under-the-curve in the presence and absence of sCD4, and data shown are averages of three independent experiments each performed with technical duplicates. *p<0.05, Wilcoxon matched-pair signed rank test.

To stabilize and select well-folded soluble BG505 SOSIP trimers we combined optimized GLA cross-linking with positive selection of correctly-folded trimers using quarternary epitope-specific gp120-gp41 interface bNAb PGT151 [78] and negative selection of subspecies that expose the immunodominant non-neutralizing gp120 V3 region [79] (Fig 1A). Quantification of free amines showed that approximately 49% were modified using this protocol (Fig 1B). Assuming 99 lysines (K) and 102 arginines (R) per trimer, this suggests approximately 98 residues may be modified by GLA treatment. The resulting selected GLA-SOSIP trimers were largely resistant to reducing SDS-PAGE, unlike the SOSIP trimer that dissociated into monomeric species (Fig 1C). Under reducing SDS-PAGE conditions a small proportion of dimers (~8%) and monomers (~3%) was observed, representing incomplete cross-linking of a minor subset of trimers. SOSIP and GLA-SOSIP trimers were compared for antigenicity by ELISA, using a large panel of mAbs previously determined to react with BG505 Env [11, 29] (Fig 1D and S1 Fig). To quantify the weak binding signal for non-NAbs, the assays were over-developed compared to bNAbs to yield where possible OD values of >1 (S1 Fig), and therefore the absolute magnitude of the bNAb and non-NAb data sets is not directly comparable. The data are expressed as a binding index, which is the ratio of ligand binding to GLA-SOSIP trimer / unmodified SOSIP trimer, where a value of 1 represents no change in binding and 0 represents complete loss of binding to cross-linked trimer. Non-NAbs globally lost reactivity (7-fold median loss of binding, Fig 1D), most likely resulting from covalent stabilization of the cross-linked ‘closed’ form of the GLA-SOSIP trimer that binds non-NAbs weakly or not at all [80]. V3-specific non-NAbs showed 2.1–3.3-fold reduced binding (median 2.7-fold) consistent with previous depletion experiments [29]. Additionally, three autologous rabbit monoclonal NAbs to the N241/N289 ‘glycan-hole’ surface [46], showed a median ~1.5-fold reduction in binding in ELISA (Fig 1D).

SPR-based binding indices derived from analysis of fragment antigen-binding (Fab) on- and off-rates confirmed and extended the results obtained by ELISA (Fig 1E and S2 Fig and S1 Table). Interestingly, V3 non-NAb 4025 showed residual binding to the GLA-SOSIP trimer. Although the binding signal was 4.7-fold lower than for unmodified protein, the KD was virtually unchanged (370 nM for SOSIP trimer, 350 nM for GLA-SOSIP trimer, see S2 Fig and S1 Table), suggesting partial depletion of trimers capable of binding 4025 with remaining trimers retaining full binding affinity. By contrast, bNAbs broadly retained reactivity significantly better than non-NAbs (1.1-fold and 1.2-fold median loss of binding by ELISA and SPR, respectively, *p*<0.0001 for ELISA, *p*<0.01 for SPR, Mann-Whitney U test). An exception to this pattern was the quaternary epitope trimer apex-specific bNAb PGT145 [71] (3.3-fold loss of binding in ELISA, 5.3-fold loss of binding in SPR). Binding of soluble (s)CD4 to SOSIP and GLA-SOSIP trimer measured by ELISA was dramatically reduced (Fig 1D and S1 Fig), consistent with the requirement for conformational changes in Env required for high-affinity CD4 binding that would be prevented by cross-linking. The exposure of CD4-induced (CD4i) and V3 loop epitopes was evaluated in the presence and absence of sCD4, and their exposure found to be prevented by cross-linking (Fig 1F and S3A and S3B Fig). We previously reported antigenic properties of GLA-SOSIP trimers with or without positive selection with PGT151 or negative selection with 19b [29]. The double antibody selection performed here resulted in a significantly improved antigenic profile compared to GLA cross-linking without selection (S3C Fig, *p*<0.01, one-way ANOVA with Dunn’s multiple comparison correction) or after PGT151 enrichment alone (*p*<0.001). By contrast, the antigenic profile of the double-selected GLA-SOSIP trimer was indistinguishable (*p*>0.999) from exclusively V3-depleted material (S3C Fig), suggesting that the depletion of V3 non-Nab-reactive trimer had the greater overall impact on antigenicity.

### GLA-SOSIP trimer structure at 4.2 Å

**Fig 2:**
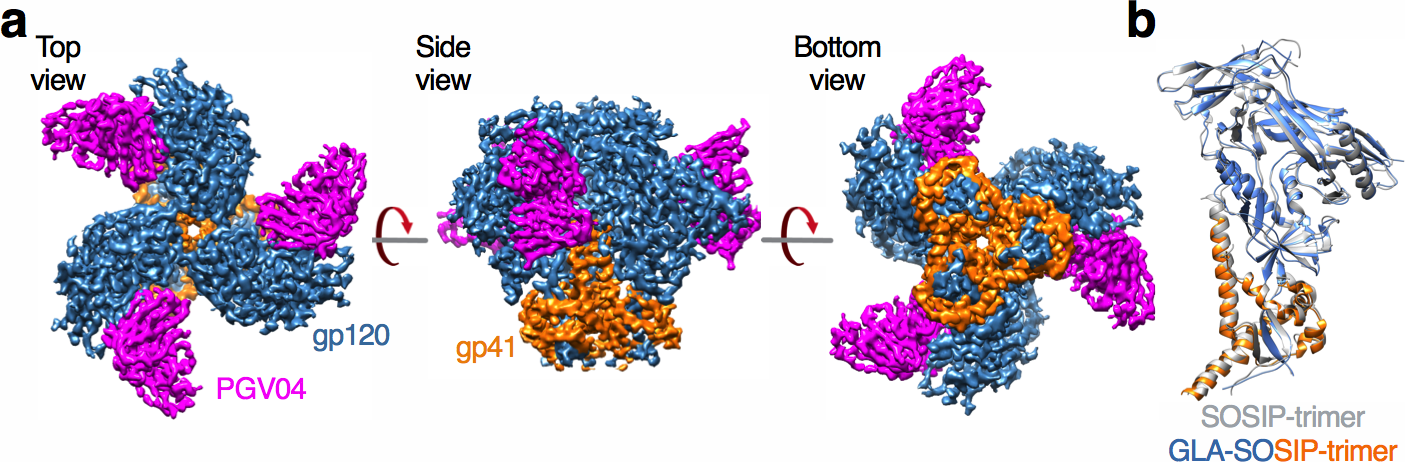
Cryo-EM model of GLA-SOSIP trimer at 4.2 Å. (**a**) Cryo-EM 3D reconstruction at 4.2 Å resolution of GLA-SOSIP trimer complexed with 3 PGV04 Fabs showing top, side and bottom views. Cryo-EM data analysis parameters are presented in S2 Table and S4 Fig. (**b**) Superposition of SOSIP (PDB ID 5CEZ) and GLA-SOSIP trimer single gp120-gp41 protomers where the unmodified SOSIP structure is in grey and the GLA-SOSIP structure is in blue (gp120) and orange (gp41). The differential Cα RMSD for the gp120-gp41 protomer is 1.48 Å.

Together these data suggested that cross-linking either induced a global distortion of the trimer fold, or more locally affected specific regions of the trimer. To probe this, we carried out cryo-EM analysis and solved a 4.2 Å resolution structure of GLA-SOSIP trimer bound to three CD4 binding site-specific PGV04 Fab fragments [81] (Fig 2A, S2 Table and S4 Fig). Overall the trimer contained the same structural features as previously described for the BG505 SOSIP trimer [13–15] and was highly similar to these structures, differing within each gp120-gp41 protomer by an RMSD based on Cα alignment of 1.48 Å (Fig 2B). Similar modest differences in RMSD were noted for the gp120 (1.45 Å) and gp41 (1.09 Å) subunits and the assembled trimer (1.50 Å). These results confirm that GLA cross-linking followed by antibody selection produced a trimer with almost identical overall structure compared to unmodified SOSIP trimer, but with improved stability and antigenicity, and strongly reduced CD4 binding and induction of CD4i and V3 loop epitopes.

### Identification of individual cross-links

**Fig 3:**
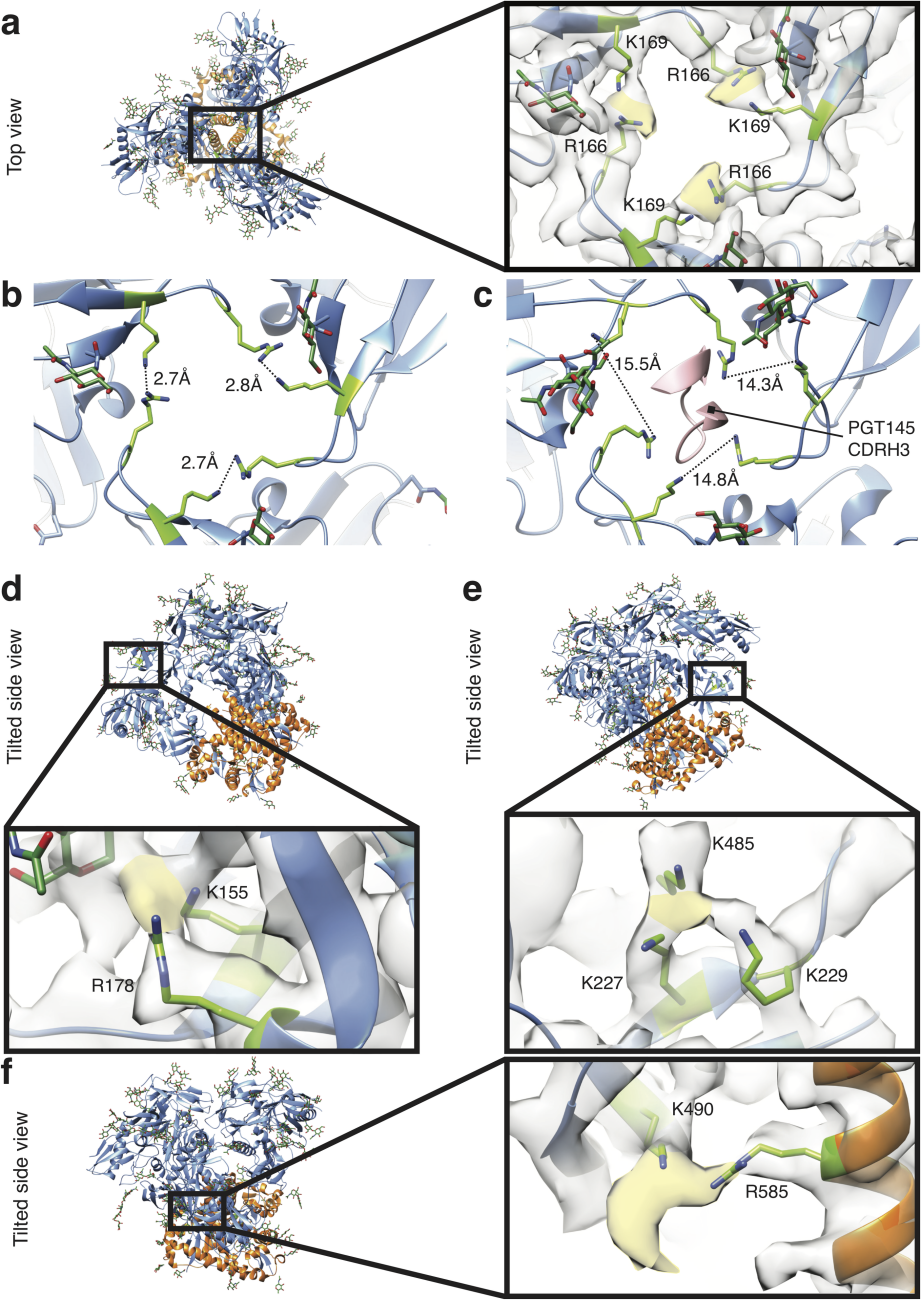
Visualization of cross-links in GLA-trimer. (**a**) Top view of the GLA-SOSIP trimer-PGV04 Fab complex with lysines (K)169 and arginines (R)166 displayed in green. Extra density corresponding to GLA crosslinks between K and R side chains is colored in yellow. (**b**) Distances between K169 and R166 side chains in the GLA-SOSIP trimer. (**c**) Distances between K169 and R166 side chains in the PGT145-bound SOSIP trimer structure (PDB ID 5V8L), with the PGT145 CDRH3 contact region shown in pink. (**d**) Tilted side view of GLA-SOSIP trimer revealing extra density (yellow) corresponding to an inter-gp120 protomer cross-link between K155 and R178. (**e**) Tilted side view of GLA-SOSIP trimer revealing cross-linking (yellow) between gp120 residues K227, K229 and K485, proximal to the BG505 N241 and N289 glycan holes. (**f**) Tilted side view of GLA-SOSIP trimer revealing extra density (yellow) corresponding to a cross-link between K490 (gp120) and R585 (gp41).

Upon close inspection of the GLA-SOSIP trimer structure, certain K and R residues were associated with additional density lacking in SOSIP trimer structures [62, 71, 82], which most likely corresponds to cross-links (Fig 3), consistent with the dominant susceptibility of these amino acids to GLA modification [83]. Of particular relevance to trimer stabilization, we observed cross-links between the V1V2 regions of adjacent gp120s by a network involving R166 and K169 at the trimer apex (Fig 3A). These modifications to the apex will trap the trimer into a stable ‘closed’ form, helping to explain the improved stability (Fig 1C) and antigenicity (Fig 1D and 1E) of GLA-SOSIP compared to SOSIP trimer. In the current structure the R166 and K169 side-chain nitrogen atoms are 2.7–2.8 Å apart (Fig 3B and Table 1), compared to 7.8 Å in a previously published structure (PDB ID 5ACO) of SOSIP trimer (Table 1). By contrast, a cryo-EM structure of BG505 SOSIP trimer in complex with PGT145 (PDB ID 5V8L) shows major rearrangement of the apex, resulting in a distance of 14.3 – 15.5 Å between R166 and K169 (Fig 3C) [71]. When comparing the arrangement of V2 residues 160–171 on all three protomers between our GLA-SOSIP trimer and the PGT145-bound SOSIP trimer, all-atom RMSD is 2.13 Å and Cα RMSD is 0.78Å. Thus, difference in V2 apex arrangement between the two structures can be attributed to both side-chain and main-chain movement. The reduced binding of PGT145 to BG505 SOSIP trimer after GLA modification (Fig 1D and 1E) is therefore probably explained by cross-linking of these residues preventing apex remodeling. An alternative explanation might be steric interference for PGT145 engagement with K169 by GLA adduction, but this is less likely since K169 is also important for binding to other V1V2 apex bNAbs such as PG16, which is minimally affected by cross-linking (S1 and S2 Figs). The V1V2 region was also cross-linked between K155 and R178 within a single gp120 monomer (Fig 3D and Table 1), but this would most likely not influence trimer stability and was not predicted to interfere with bNAb binding due to the distance between these residues and the epitopes of known V1V2 bNAbs [15, 84]. We observed a cluster of intra-gp120 cross-linked lysines (K227, K229 and K485, Fig 3E) within the recently described N241/N289 glycan hole NAb epitope that is a common target of autologous NAbs in SOSIP trimer-immunized rabbits [19, 46]. This is consistent with the reduced binding of the N241/N289 glycan-hole targeting autologous NAbs (Fig 1D and S1 Fig), and indicates a binding mode partially disfavored by GLA addition. Further cross-links contributing to trimer stability were present between gp120 and gp41 subunits within a single protomer, in which HR1 of gp41 (R585) was cross-linked to gp120 residue K490 in β29 of gp120 (Fig 3F, Table 1). Binding of gp41/gp120 interface-directed bNAbs, including PGT151, 3BC315, 3BC317 and 35O22, was unchanged or only subtly reduced in GLA-SOSIP trimer (S1 Fig), which was expected as none of these mAbs form contacts proximal to K490 or R585 [73, 78]. In summary, chemical cross-links visualized for the first time within a vaccine antigen were associated with specific K and R residues in the trimer that stabilized inter-subunit interactions and influenced bNAb binding in a highly epitope-specific manner.

**Table 1:** Observed cross-links in GLA-SOSIP trimer.

Residues	Distance (Å) SOSIP trimer	Distance (Å) GLA-SOSIP trimer	Location
K155 <-> R178	7.8	2.4	Apex, intra-gp120
R166 <-> K169	7.8	2.7	Apex, inter-protomer
K227 <-> K229	5.8	6.0	Intra-gp120
K227 <-> K485	5.8	5.4	Intra-gp120
K229 <-> K485	9.3	4.4	Intra-gp120
K490 <-> R585	6.2	2.9	Inter-gp120-gp41

### Analysis of GLA-mediated cross-links

GLA is reported to link K or R side-chain amines that are ~7–10 Å apart in proteins [85], and results in a range of cross-linked distances consistent with the diversity of species present within aqueous GLA solution: monomeric linear, cyclic and polymeric [83]. Shorter cross-links may be made by cyclic GLA forms, whereas longer cross-links are predicted to result from linear GLA cross-linking [85, 86]. In the current structure, the distances measured between the closest pair of side chain nitrogen (N) atoms of the GLA modified K-K (ND2 –ND2) and K-R (ND2 –NE or ND2 –NH1 or ND2 –NH2) pairs ranged from 2.4 to 6.0 Å, whereas in a previously reported unmodified BG505 SOSIP trimer structure (PDB ID 5ACO) [82] the same side-chain pairs were separated by 5.8 to 9.3 Å (Table 1)**.** The finding that cross-linked side chains are closer than their unmodified counterparts probably accounts for the RMSD differential between SOSIP- and GLA-SOSIP trimer, and is consistent with previous estimates of K-K distances after inter-molecular GLA cross-linking within hen egg lysozyme crystal lattices [86, 87]. We note that several other K-K and K-R pairs are separated by similar distances in SOSIP trimer and are therefore potentially sensitive to GLA cross-linking, but are not obviously cross-linked in the GLA-SOSIP trimer density map (Table 2). It is unclear if these residues were modified inefficiently by GLA and therefore corresponding cross-links of partial occupancy were not visible in the averaged structure, or whether GLA modified the residues efficiently without generating a well-ordered cross-link, or no modifications were made at these sites.

**Table 2:** Unobserved potential cross-linking targets in GLA-SOSIP trimer.

Residues	Distance (Å) SOSIP trimer	Distance (Å) GLA-SOSIP trimer
K46 <-> K490	9.2	7.3
K46 <-> K633	8.8	14.9
K97 <-> K282	10.0	7.9
K117 <-> K117	6.0	10.2
K121 <-> K121	8.6	8.3
K231 <-> K232	10.0	8.0
K232 <-> K351	7.0	7.0
K344 <-> K347	11.5	7.0
R503 <-> K655	19.9	8.2

### Binding antibody responses to trimer immunization

**Fig 4:**
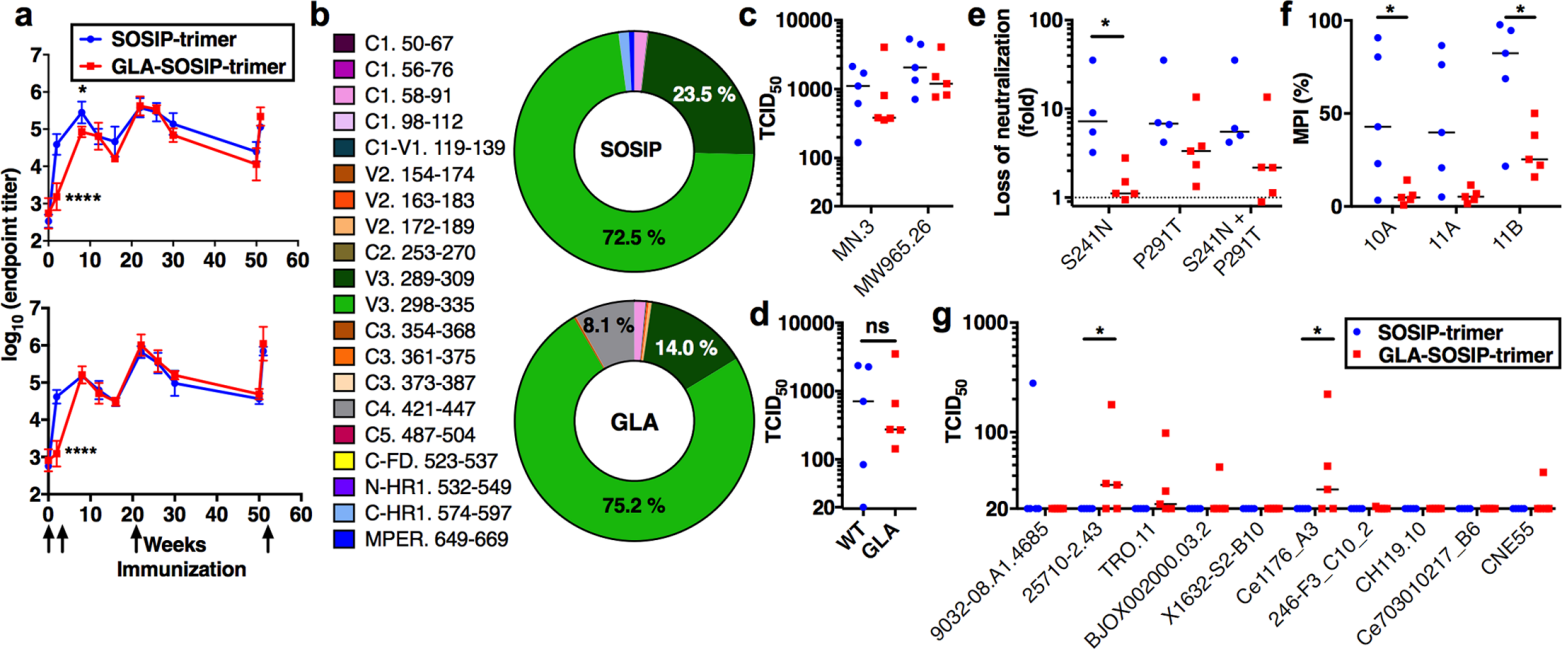
Rabbit antibody responses to SOSIP and GLA-SOSIP trimer. (**a**) ELISA endpoint titers of rabbit sera binding to SOSIP (top panel) and GLA-SOSIP trimer (bottom panel). Arrows represent immunizations at weeks 0, 4, 20 and 50, datum points are means of 5 animals/group and error bars indicate SD. *p<0.05; ****p<0.0001, One-way ANOVA with Sidak’s multiple-comparison correction. (**b**) Linear peptide array mapping of rabbit serum responses, pie charts represent proportion of response to individual epitopes with the region for each epitope listed with dominant percentage reactivity shown. Values plotted are magnitude of binding to any strain among the 13 strains in the array library for each epitope. Regions and sequences listed on left. Supporting data in S5 Fig. (**c, d**) Neutralization of (**c**) neutralization-sensitive Tier-1 PV and (**d**) autologous Tier-2 PV by sera from SOSIP and GLA-SOSIP trimer-immunized rabbits. Supporting data in S6 Fig. (**d**) ns = not significant, Mann-Whitney U test. (**e**) Loss of neutralization activity of SOSIP relative to GLA-SOSIP trimer sera on mutant PV. (**f**) Cross-competition ELISA of sera with the indicated glycan-hole specific mAbs is shown as maximum percent inhibition (MPI). *p<0.05, One-way ANOVA with Sidak’s multiple-comparison correction. (**g**) Neutralization of heterologous Tier-2 PV, ns = not significant, *p<0.05, Kruskal-Wallis test with Dunn’s multiple-comparison correction.

Since no previous studies have interrogated high-resolution structural and immunogenic features of the same unmodified and cross-linked vaccine antigen, we compared SOSIP with GLA-SOSIP trimer immunogenicity. Antigens were formulated in Iscomatrix adjuvant to allow direct comparison with prior BG505 SOSIP trimer-based immunogenicity studies [16, 17, 19, 29, 88]. To assess antibody responses, rabbits were immunized using an extended regimen at 0, 4, 20 and 50 weeks with homologous antigen and adjuvant in series, and sera assayed by ELISA for binding IgG responses to SOSIP or GLA-SOSIP trimer (Fig 4A) captured onto the solid-phase via the glycan-reactive bNAb 2G12 [29]. Antigen-specific IgG, presented as means of individual sera ± SD, was detectable in both groups at 2 weeks post-prime, but the endpoint titers in the GLA-SOSIP trimer-immunized group were significantly (average 25-fold, *p*<0.0001 one-way ANOVA with Sidak’s post-test) lower than in the SOSIP trimer group. We hypothesize that this difference in immunogenicity may reflect reduced T helper (Th) cell responses to the cross-linked trimer (see below). However, at week 22 after the first boost this difference was eliminated when sera were titrated on GLA-SOSIP, or reduced (3.3-fold, *p* = 0.033) on unmodified SOSIP trimer, and comparison of subsequent time points revealed no significant differences between groups. Titers declined in both groups from peaks of ~10^5.5^ (assayed on SOSIP trimer) and ~10^5.9^ (assayed on GLA-SOSIP trimer) after the week 22 second boost to ~10^4.3^ (assayed on SOSIP trimer) and ~10^4.5^ (assayed on GLA-SOSIP trimer) at week 50, then increased after the 3^rd^ boost by ~10-fold at the final week 51 bleed. Thus, apart from the early post-prime response, BG505 Env elicited similar global trimer binding antibody responses whether SOSIP or GLA-SOSIP, or assayed on SOSIP or GLA-SOSIP material, implying the absence of immunodominant GLA-elicited neo-epitopes.

### Specificity and neutralizing activity of trimer-elicited antibodies

To analyze antibody specificity, we first tested binding to a series of overlapping peptides spanning Env and covering multiple viral sequences of different global origins by peptide array [76]. As observed in previous immunization analyses [76], the V3 region was highly immunodominant, eliciting the majority of antibody responses recognizing linear epitopes (Fig 4B, S5 Fig). The proportion of antibodies binding V3 in the GLA-SOSIP trimer group was modestly reduced compared to SOSIP trimer, replaced mainly by C4-specific responses, showing that depletion of V3-exposed cross-linked species only marginally influenced the immunogenicity of this domain to induce linear peptide-binding antibodies. Serum responses to linear epitopes in gp41 HR1 and MPER were observed in SOSIP but not GLA-SOSIP trimer immunized rabbit sera (S5 Fig), implying GLA-mediated masking of gp41 elements, possibly by overall trimer stabilization.

Neutralization activity of the week 51 sera was measured using a panel of HIV-1 pseudoviruses (PV) carrying highly neutralization-sensitive Tier-1 or more difficult-to-neutralize Tier-2 Envs [89] (Fig 4C, 4D and 4G and S6 Fig). Sera from SOSIP trimer gave higher median reciprocal 50% inhibition titers of both Tier-1 PVs tested (~1/1000 and ~1/2000) compared to GLA-SOSIP trimer (1/380 and ~1/1000) for MN.3 and MW965.26 respectively, but these differences were not significant (Fig 4C and S6 Fig). Nevertheless, since most Tier-1 neutralizing activity is mediated by V3-specific antibodies [79, 90], this result is broadly consistent with the peptide mapping, and demonstrates that GLA cross-linking and depletion of V3 epitope-expressing trimers is insufficient to prevent B cell recognition of this immunodominant region.

Similar to previous studies [16, 46], all but one animal developed NAbs against the autologous BG505 PV with mean TCID_50_ titers of ~1/800 and ~1/300 for SOSIP and GLA-SOSIP trimer respectively, a non-significant difference (Fig 4D). Autologous NAb responses in SOSIP trimer-immunized rabbits frequently target a protein surface, termed a ‘glycan hole’, exposed by loss of N-linked glycans at positions 241 and 289 [19, 46]. To interrogate this, we probed serum neutralizing epitope specificity using PV in which the glycosylation sequons at either or both of these positions were restored. Consistent with previous studies [19, 46], restoration of either or both glycans substantially reduced neutralizing titers (median loss 6.8-, 7.2- and 5.5-fold for position 241, 289 and 241+289, respectively) with sera from SOSIP trimer immunized animals (Fig 4E). By contrast, animals immunized with GLA-SOSIP trimer neutralized PV with restored glycans with minimal reduction in neutralization (median loss 1.1-, 3.4- and 2.2-fold at positions 241, 289 and 241+289 respectively). Indeed, the GLA-SOSIP trimer sera gave a significantly (*p* = 0.0241 one-way ANOVA with Kruskal Wallis’ post-test) smaller effect than observed in animals immunized with SOSIP trimer on the S241N mutant PV (Fig 4E). We confirmed modulation of serum specificity to these regions by performing cross-competition of sera with known glycan-hole targeting mAbs^45^. Sera from the SOSIP trimer group showed significantly stronger glycan-hole specific responses than those from GLA-SOSIP trimer immunized animals (Fig 4F) for mAbs 10A (*p* = 0.041; 1 way ANOVA with Sidak’s post-test) and 11B (*p* = 0.038) and a similar, though not statistically significant (*p* = 0.057) trend for 11A. These findings are of interest because cross-links were detected proximal to the BG505 glycan hole NAb binding epitopes (Fig 3E), suggesting modulation of B-cell recognition consistent with the observed reduction in binding of mAbs targeting this region in vitro (Fig 1D and S1 Fig).

Potent neutralization of difficult-to-neutralize heterologous Tier-2 viruses that are representative of circulating clinical isolates is the principal goal of HIV-1 antibody-based vaccine design. We therefore tested a global reference panel of 10 Tier-2 PV from different geographic regions and of diverse clades [74], and noted greater neutralization in sera from the GLA-SOSIP trimer compared to SOSIP trimer-immunized groups (Fig 4G and S6 Fig). Whereas sera from only one SOSIP trimer-immunized rabbit neutralized a single Tier-2 PV (9032-08.A1.4685, also known as B41), sera from 3/5 GLA-SOSIP trimer-immunized rabbits neutralized a cumulative total of 6 heterologous PV with a range of 1/21–1/222 (Fig 4G and S6 Fig). One serum (rabbit 1721) neutralized 6 PVs whereas two other sera (rabbits 1717 and 1720) neutralized 3 PVs. For PV 25710–2.43 and Ce1176_A3, neutralization was significantly higher in the GLA-SOSIP trimer group compared to the SOSIP trimer group (*p* = 0.049 and 0.040, respectively; Kuskal-Wallis test with Dunn’s post-test). We have previously observed sporadic weak heterologous Tier-2 PV neutralization by immune rabbit sera after BG505 Env immunization using protein-protein [26, 91] or vector-protein [91] prime-boost regimens, or heterologous SOSIP trimers administered in series or parallel [19]. However, under these conditions 50% inhibition titers failed to reach [91] or rarely exceeded [19, 26] 1/100 in individual sera. At present we do not know the specificity of these heterologous Tier-2 neutralizing antibody responses but this is currently under analysis. A very recent study using optimized administration protocols and stabilized or liposome-formulated Env trimers in macaques attained 50% serum inhibition titers of up to ~1/300 against 3/10 PV from the global panel [88]. Thus, whilst titers of heterologous Tier-2 neutralization that we observed in the current study using homologous GLA-SOSIP trimer immunizations were also relatively modest, it is likely that these could be enhanced with improved immunization regimens.

In summary, relatively subtle and highly localized differences were observed in the titer and specificity of antibody responses to SOSIP and GLA-SOSIP trimers, but autologous Tier-2 neutralization specificity was modified and heterologous neutralizing activity was broadened in animals immunized with cross-linked trimers.

### Th cell responses to SOSIP and GLA-SOSIP trimers

**Fig 5:**
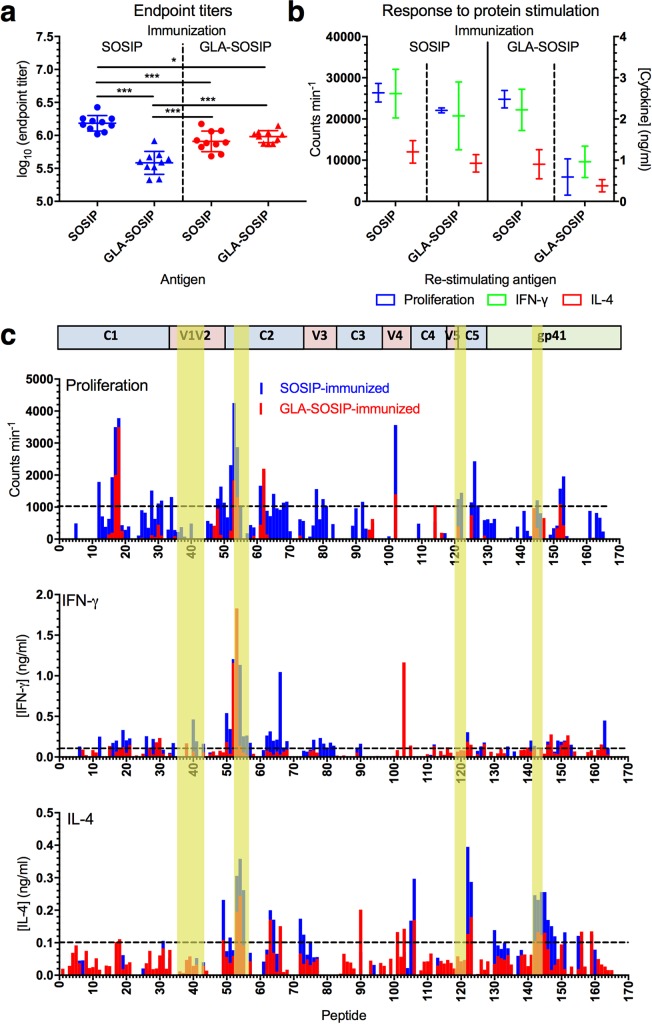
Murine antibody and CD4 Th cell responses to GLA-SOSIP trimer. (**a**) Endpoint titers of week 8 sera from SOSIP or GLA-SOSIP trimer-immunized mice. Datum points represent individual mouse sera (n = 10) from two independent experiments, and bars represent median ± SD. X-axis represents ELISA coating antigen. ***p<0.001 Mann Whitney U. (**b**) CD4 T cells negatively isolated from splenocytes at week 8 post-prime were re-stimulated in vitro with SOSIP or GLA-SOSIP trimer and assayed for proliferation, IFN-γ or IL-4 production. Data represent readouts from pooled Th cells from each mouse group merged from two independent experiments, bars represent median ± SD. (**c**) CD4 T cells negatively isolated from splenocytes at week 8 post-prime were pooled and re-stimulated in vitro using an overlapping 15-mer BG505 peptide library (peptides 1–165), and assayed for Th cell proliferation (top panel), IFN-γ production (middle panel) and IL-4 production (lower panel). Data are averaged from two independent experiments. Horizontal bar represents secondary structure regions of BG505. Gold bars represent regions in which cross-links were identified in the GLA-SOSIP trimer structure, and dotted black lines represent assay cutoff. Supporting data in S7 and S8 Figs.

Because Th cell responses to vaccination are critical for B cell activation of class switching and somatic hypermutation required for antibody affinity maturation, we set about characterizing these for SOSIP and GLA-SOSIP trimers. Since immunological reagents are not available for analysis of rabbit T cell responses, we immunized BALB/c mice with SOSIP and GLA-SOSIP trimer in Iscomatrix to parallel the rabbit immunizations. Following a prime and boost to develop and mature Th cell memory responses, sera and spleens were harvested at week 4 post-boost and CD4 Th cells isolated. Serum endpoint IgG titers were reduced in GLA-SOSIP trimer compared to SOSIP trimer-immunized animals when assayed on SOSIP trimer (Fig 5A; *p*<0.001, one way ANOVA), most likely reflecting reduced exposure of immunodominant non-neutralizing epitopes on GLA-SOSIP trimer. When assayed on GLA-SOSIP trimer, titers were reduced in the SOSIP trimer-immunized group compared to the GLA-SOSIP trimer group, again probably reflecting induction of immunodominant non-NAbs by SOSIP trimer, that fail to bind GLA-SOSIP trimer. CD4 Th cells were re-stimulated in vitro with SOSIP or GLA-SOSIP trimer, and T cell proliferation and IFN-γ and IL-4 production, representing Th type-1 (Th1) and type-2 (Th2) responses respectively, were assessed. Th cells from SOSIP-trimer immunized mice gave similar proliferation and cytokine responses whether re-stimulated with SOSIP or GLA-SOSIP trimer (Fig 5B). By contrast, mice immunized with GLA-SOSIP trimer showed a trend towards more robust proliferation (~4-fold) and cytokine (~2-fold) responses to SOSIP than to GLA-SOSIP trimer re-stimulation, suggesting that cross-linked trimer may be less efficiently processed and/or presented in vitro to GLA-SOSIP trimer than to SOSIP trimer-primed memory T cells.

To characterize the epitope specificity of Th cell epitopes recognized by mice immunized with SOSIP or GLA-SOSIP trimer, recall proliferative and cytokine responses were mapped in Th cells pooled from mice within each group using an overlapping BG505 Env 15-mer peptide library (Fig 5C and S7 and S8 Figs). Stringent criteria were applied to the identification of Th epitopes, requiring responses to 2 or more overlapping peptides with positive responses in two independent experiments and a cutoff of >1000 counts per minute (CPM) for proliferation, and positive responses in both experiments and a mean response of >0.1 OD for IFN-γ and IL-4 secretion. Immunodominant proliferative responses were observed in the group immunized with SOSIP trimer at peptide positions 17–18 (amino acids, aa 65–83 corresponding to gp120 C1), 53–54 (aa 209–227, gp120 C2), 61–62 (aa 241–259, gp120 C2) and 68–69 (aa 269–287, gp120 C2). These epitopes fell into 3 major epitope clusters within gp120 C1 or C2, and one more minor epitope also within gp120 C2 (S8 Fig). Of these epitope-specific responses primed by SOSIP trimer, only peptides 17–18 (aa 65–83) retained full restimulation activity in the GLA-SOSIP trimer immunized mice, revealing partial or complete loss of immunodominant epitopes at the other epitope regions (Fig 5C and S7 and S8 Figs). Interestingly, aside from the epitope at peptides 53–54 (aa 209–227) that contains a cross-linked residue K227 as defined in Fig 3E, the other epitopes do not contain visible cross-links. This suggests that although cross-linking may affect the 209–227 epitope directly, other epitopes are probably influenced by amine modification on K and R (Fig 1B) that interferes with MHC class-II binding or T cell receptor recognition. Additionally, cross-linking and amine modification may indirectly influence Th cell activation via effects on protein processing and peptide loading into MHC class-II as proposed for cross-linked model antigens [92, 93]. IFN-γ responses were within the same major epitope clusters 1–3 as the proliferative responses, although in several cases shifted by one or more peptides (Fig 5C and S7 and S8 Figs). As with proliferative responses, the number of IFN-γ responses to GLA-SOSIP trimer-immunization was reduced compared to SOSIP trimer immunization, but was nevertheless maintained at several sites (Fig 5 and S7 and S8 Figs) and de-novo responses were observed at five epitopes (S8 Fig). IL-4 responses to SOSIP trimer immunization were observed in major epitope clusters 2, 3, and 4, and three other more minor regions (Fig 5 and S8 Fig). Several of these responses were lost and a new specificity gained after GLA-SOSIP trimer immunization (Fig 5 and S7 and S8 Figs).

In summary, major Th cell epitopes within SOSIP trimer were primarily located within 5 conserved regions in gp120 C1 and C2 and gp41 HR1, with other contributions from gp120 C4/V4 and C5 (Fig 5 and S7 and S8 Figs). Immunization with GLA-SOSIP trimer showed a trend towards reduced epitope-specific Th cell recall proliferative and cytokine responses with generation of de-novo cytokine responses not elicited by SOSIP trimer, but with no obvious skewing of Th polarization.

## Discussion

Despite having been successfully used in millions of vaccine doses for almost 100 years, chemical cross-linking technology continues to be applied empirically without comparison of structural or immunological impacts of the antigen with its unmodified counterpart. Here, we demonstrate for the first time that cross-linking and antibody selection of a highly conformational, vaccine-relevant HIV-1 Env-based glycoprotein trimer yields a well-folded product that is structurally almost identical to its unmodified counterpart. The cross-linked trimer had improved antigenicity in terms of reduced non-Nab binding with largely retained bNAb binding, but CD4 binding and V3 loop and CD4-induced epitope exposure were almost completely abrogated. These latter points are particularly relevant to human immunization, since binding to CD4 on T cells would sequester unmodified trimer, obscure the CD4 binding site from B cell recognition, and expose non-neutralizing immunodominant epitopes for B cell recognition [94]. Although the cross-linked and unmodified trimers elicited similar global antibody immune responses, some striking specific differential responses were observed, the most important of which were modest but significant broadening of heterologous Tier-2 neutralization and modified specificity of autologous Tier-2 neutralization in the GLA-SOSIP trimer-immunized group. Whilst heterologous Tier-2 neutralization titers were not high, they were nevertheless similar to, or exceeded, those in previous rabbit immunization studies using soluble next-generation Env trimers stabilized with SOSIP or other mutations [19, 26]. Further structural and immunologic analysis of cross-linked, antibody-selected BG505 trimers will yield clues as to the specificity of Tier-2 neutralization, and improved immunization strategies will seek to enhance NAb titers [88, 95].

Antigen stabilization can optimize induction of protective antibodies, as demonstrated for Respiratory Syncytial virus fusion (F) glycoprotein [96]. However, although increased stability of soluble HIV-1 Env trimers correlated in one study with enhanced Tier-2 neutralization activity [18], in other studies it did not [17, 88]. Therefore, simply enhancing HIV-1 trimer stability may be insufficient to elicit bNAbs, and presentation to the immune system of specific stabilized conformational species of Env, as here, may be required. That the broadening of Tier-2 neutralization by cross-linking trimer immunogens in the current study was only modest is not surprising, since HIV-1 Env has evolved multiple antibody evasion mechanisms additional to conformational instability, including extreme antigenic variability and an extensive glycan shield. Thus, enhancing trimer stability is only one element of vaccine immunogen design that will need to be considered alongside other approaches. Nevertherless, our findings suggest that chemical cross-linking followed by quaternary-epitope antibody selection of specific ‘locked’ conformational species is a concept with particular promise for HIV-1 neutralizing antibody-based immunogen design that may also be generally applicable to design of other vaccines.

Aside from optimal presentation of bNAb epitopes, it has been hypothesized that B cell clones elicited by immunodominant regions (particularly V3 and the trimer base) might compete for T cell help with rare bNAb-producing B cells [97], reducing bNAb elicitation. Although we failed to significantly reduce V3-directed antibody responses as indicated by linear peptide binding and Tier-1 neutralization, evidence supporting the importance of V3 immunogenicity suppression to promote bNAb responses is currently lacking. Several studies have shown that reduced or absent V3 antibody induction by immunization correlates neither with increased B cell responses to other specificities [98], nor with NAb potency or breadth [17, 79, 88]. Moreover, bNAb responses arise in HIV-1-infected individuals in the presence of strong non-neutralizing, including anti-V3, antibody responses [10].

Induction of bNAbs will require Th cell help [99, 100], and to probe this we analyzed Th responses to WT- and GLA-trimers in mice. Whilst murine Th responses will differ in repertoire with respect to human responses, these data are nevertheless helpful in defining whether cross-linking will modify their relative magnitude and/or specificity. Few previous studies have interrogated Th responses to HIV-1 Env glycoproteins, and none has previously investigated responses to immunization with correctly-folded vaccine-relevant soluble trimers such as BG505 SOSIP. Nevertheless, our finding that most Th responses are located primarily within conserved gp120 C1 and C2 regions is consistent with previous findings [101, 102]. Whilst protein stabilization may promote the immunogenicity of conformational B cell epitopes, the converse may be true of Th cell epitopes since introduction of additional covalent bonds reduces antigen processing in the MHC class-II compartment, reducing peptides available for presentation to T cells, and potentially modifying the peptide repertoire presented [93]. Moreover, the estimated 49% of amine modification of K and R side chains in GLA-SOSIP trimer is likely to impact upon MHC class-II presentation and/or T cell receptor recognition even in the absence of cross-links. Although the similar levels of binding antibody after boosting, and enhanced heterologous Tier-2 neutralizing responses that we observed in the GLA-SOSIP trimer-immunized group suggest that Th responses are not limiting in this context, priming of more robust Th responses with heterologous immunogens such as via ‘intrastructural help’ [103] may bring further benefit. Aldehydes have a long history of use in vaccines and are therefore safe for use in man as inactivating and cross-linking agents [1]. Whilst GLA generally improved BG505 SOSIP trimer B cell antigenicity, the reduction in PGT145 bNAb binding and reduced Th cell responses suggest that it may not be optimal in this setting. Moreover, aldehydes adduct carbonyl groups to proteins that may have a Th2-type skewing effect [104], as has recently been shown for HIV-1 Env trimers in mice [105]. Although our current analysis revealed a general dampening of Th responses, we did not observe any obvious skewing from Th1 towards Th2.

Some general structural principles relevant to vaccine design may be extracted from our analysis. We observed cross-links between K and/or R residues that were ~6–9 Å apart in the unmodified structure, consistent with the predicted dimensions of the linear GLA monomer (~7.5 Å). Similarly, Salem et al [85] observed cross-linking of K with R side chains ~7–9.5 Å apart in unmodified protein, implying that this range is optimal for GLA cross-linking [85]. Interestingly however, in both analyses GLA cross-links reduced N–C distances to below 6 Å, implying ‘short’ cross-links introduced potentially by non-linear forms of GLA that generate highly localized structural distortions that do not obviously perturb overall antigenicity and immunogenicity. It is tempting to speculate that the information derived from our analysis might allow prediction of preferential cross-links that would form in other proteins on the basis of their K–R distances. However, there are limitations in the current study that reduce its predictive power, including: i) the limited resolution of the structural analysis; ii) conformational flexibility intrinsic to the HIV-1 trimer that will introduce variability into K–R distances during cross-linking; iii) the heterogeneity of GLA in solution, with linear and cyclic monomeric, dimeric and oligomeric species having been reported [83, 86, 87]. In this latter respect, use of synthetic cross-linking reagents that are homogeneous with a well-defined cross-linking distance would improve the predictive power of cross-linking. In a previous study we tested the hetero-bifunctional cross-linker 1-Ethyl-3-[3-dimethylaminopropyl] carbodiimide hydrochloride (EDC), and reported enhanced trimer stability without loss of apex-specific bNAb binding [29]. Others have used chemical cross-linkers (bis (sulfosuccinimidyl) suberate (BS^3^) and 3,3′-dithiobis (sulfosuccinimidylpropionate) (DTSSP) for membrane–anchored trimer stabilization and reported favourable stability and antigenic properties after affinity selection of well-folded species [28]. Thus, further analysis of different cross-linkers and testing of different antibodies for cross-linked trimer selection may lead to additional improvements in immunogen design.

Taken together, our data define for the first time the location of stabilizing cross-links within a complex and highly conformational HIV-1 vaccine antigen, which mediate modest improvements in neutralizing antibody breadth. This information will pave the way for more rational use of chemical cross-linking as a targeted approach to antigenic stabilization and selection of beneficial conformational species in vaccine design against HIV-1 and other pathogens.

## Supporting information

S1 Table SPR fit parameters and signal ratios. Associated binding curves are shown in S2 Fig.
(PDF)

S2 Table Cryo-EM data collection and analysis. Workflow is shown in S4 Fig and associated models are shown in Figs 2 and 3.
(PDF)

S1 Fig Binding of human non-NAbs, bNAbs, rabbit mAbs and sCD4 to SOSIP and GLA-SOSIP trimer assayed by ELISA. Binding curves of (**a**) human non-NAbs, bNAbs and sCD4 or (**b**) rabbit autologous NAbs binding to SOSIP trimer, GLA-SOSIP trimer or BSA (neg) as measured by capture ELISA. ELISA reactions were over-developed for non-NAbs to yield quantifiable binding curves. Curves shown are representative of 2–4 independent experiments. Error bars indicate SD of technical replicates.
(PDF)

S2 Fig Raw SPR traces. SOSIP-trimer (left) or GLA-SOSIP-trimer (right) were captured onto each flow cell using mAb 2G12 (not shown), and 4-fold dilution series of the indicated Fab starting at 10 μM were passed over the surface followed by buffer only. Vertical lines indicate start and stop of injection. Black curves indicate the fit of a 1:1 binding model, fit values are summarized in S1 Table and results summarized in Fig 1E.
(PDF)

S3 Fig Extended antigenic properties of cross-linked SOSIP-trimers. (**a**) Binding of V3-specific (top row) and sCD4-induced (middle row) non-NAbs to SOSIP and GLA-SOSIP trimer was assayed by capture ELISA. 2G12 (bottom) served as a loading control. Binding curves to SOSIP trimer, GLA-SOSIP trimer or BSA (neg) are shown in presence (solid symbols) and absence (empty symbols) of sCD4. ELISA reactions were over-developed for non-NAbs to yield OD values where possible >1 to allow quantification. Curves shown are representative of 3 independent experiments, error bars indicate SD of technical repeats. (**b**) sCD4-induced non-Nab binding. Binding of CD4-inducible (CD4i) and V3 non-nAbs to SOSIP-trimers (blue) and GLA-SOSIP-trimers (red) and loading control mAb 2G12 was measured in the presence (filled symbols) or absence (empty symbols) of sCD4 by ELISA. AUC values are defined as background-subtracted area under the curve, data derived from a representative experiment of two independent repeats. (**c**) Comparison of antigenic profiles of double-selected GLA-SOSIP trimers with previously published [29] unselected and V3-negative or PGT151-positive single-selected cross-linked trimers. bNAbs are shown as dark-colored filled symbols and non-NAbs as light-colored empty symbols. Data are averages of 2–6 independent repeats. **p <0.01, ***p<0.001, ns = not significant, one way ANOVA with Dunn’s multiple correction.
(PDF)

S4 Fig Cryo-EM work flow. (**a**) Flow diagram of steps utilized in cryo-EM data processing. The final reconstruction contained 55,563 molecular projection images and was obtained imposing C3 rotational symmetry during refinement. (**b**) Plot of Fourier shell correlation (FSC) between two independently refined data half sets that were combined into the final reconstructed density map. Globally averaged resolution measured by the 0.143 criterion was 4.2Å. (**c**) Plot showing angular coverage by the 55,563 molecular projection images that contributed to the final reconstructed density map.
(PDF)

S5 Fig Serum peptide mapping on overlapping multiclade 15mer linear peptides. (**a**) Serum from WT-trimer-immunized rabbits assayed on gp120 peptide array represented as fluorescent signal intensity. (**b**) Serum from GLA-trimer-immunized rabbits assayed on gp120 peptide array. (**c**) Serum from SOSIP trimer-immunized rabbits assayed on gp41 peptide array. (**d**) Serum from GLA-SOSIP trimer-immunized rabbits assayed on gp41 peptide array. Colored lines represent different clades from which peptides were derived.
(PDF)

S6 Fig Neutralization data. 50% inhibitory dilutions (TCID_50_s) are shown as determined by TZM-Bl assay. Values are colored according to their value with stronger neutralization in darker shades. Assay cutoff is a serum dilution of 1/20.
(PDF)

S7 Fig CD4 T cell peptide mapping data. CD4 T cells negatively enriched from BALB/c splenocytes were restimulated in vitro using a 165 peptide library each of 15 amino acids overlapping by 5. Data presented are from two independent experiments. The peptide number is shown alongside the relevant amino acids and the region of gp140 represented. Gold coloring represents the regions in which GLA cross-links were detected in the GLA-SOSIP trimer structure. Proliferation was measured by ^3^H incorporation, where green = response below baseline, white (1-1000cpm), pink (1001-5000cpm), red (5001-8000cpm). Thick vertical blue bars represent dominant epitope responses (mean >1000) to SOSIP trimer immunization with at least 2 positive adjacent peptide responses in both experiments, thin vertical blue bars represent single peptide positive responses with CPM >1000. Thick vertical red bars represent dominant epitope responses (mean >1000) to GLA-SOSIP trimer immunization, thin vertical red bars represent single peptide positive responses. IFN-γ was assayed by ELISA, where green = response below baseline, white (0.001–1 OD), pale pink (1.001–2 OD), red (2.001–3 OD). IL-4 was assayed by ELISA, where green = response below baseline, white (0.001–0.2), pale pink (0.2001–0.4), red (0.4001–0.6). Criteria for selecting IFN-γ and IL-4 responses were positive responses in both experiments and a mean response across both experiments of >0.1 OD. Vertical blue and red bars represent epitopes eliciting cytokine responses as defined for proliferation.
(PDF)

S8 Fig Summary of Th epitope responses to immunization with SOSIP and GLA-SOSIP. Data summarized from S7 Fig. Grey shading represents the 5 major epitope clusters (labeled 1–5) observed for Th cell proliferation and/or IFN-γ and/or IL-4 production.
(PDF)
