# Supplementary material for: Structural and immunologic correlates of chemically stabilized HIV-1 envelope glycoproteins
Source: PLoS Pathog. 2018 May 10;14(5):e1006986. doi: 10.1371/journal.ppat.1006986 (PMC5944921; doi:10.1371/journal.ppat.1006986)
Supplement: S1 Table — Associated binding curves are shown in S2 Fig. (PDF) [file ppat.1006986.s001.pdf]

Fab	SOSIP-trimer				GLA-SOSIP-trimer			
	**KD (M)**	**Kon (1/Ms)**	**Koff (1/s)**	**Signal Ratio**	**KD (M)**	**Kon (1/Ms)**	**Koff (1/s)**	**Signal Ratio**
**PGT121**	1.0E-08	9.8E+03	9.8E-05	0.37	6.2E-09	1.1E+04	6.8E-05	0.37
**PGT128**	Not 1:1 binding			0.54	Not 1:1 binding			0.60
**39F**	Low signal			0.06	No signal			0
**4025**	3.7E-07	2.0E+03	7.4E-04	0.37	3.4E-07	2.6E+03	8.7E-04	0.08
**PGT145**	Not 1:1 binding			1.57	Not 1:1 binding			0.29
**PG16**	Not 1:1 binding			1.16	Not 1:1 binding			0.99
**PGT151**	Not 1:1 binding			1.48	Not 1:1 binding			0.58
**3BC315**	7.3E-09	1.6E+04	1.2E-04	1.05	8.9E-09	1.6E+04	1.4E-04	0.78
**35O22**	5.2E-08	9.3E+03	4.8E-04	1.32	7.2E-08	9.9E+03	7.1E-04	0.95
**8ANC195**	2.4E-08	1.9E+03	4.6E-05	1.05	3.3E-08	1.8E+03	6.1E-05	0.97
**VRC01**	Off-rate out of range			1.05	Off-rate out of range			0.91
**PGV04**	Off-rate out of range			1.08	Off-rate out of range			0.97
**NIH45-46**	2.7E-10	7.5E+03	2.0E-06	1.20	2.3E-09	8.0E+03	1.8E-05	1.16
**3BNC60**	Off-rate out of range			1.20	Off-rate out of range			1.18
**B6**	Low signal			0.04	No signal			0
