# Supplementary material for: Structural and immunologic correlates of chemically stabilized HIV-1 envelope glycoproteins
Source: PLoS Pathog. 2018 May 10;14(5):e1006986. doi: 10.1371/journal.ppat.1006986 (PMC5944921; doi:10.1371/journal.ppat.1006986)
Supplement: S2 Table — Workflow is shown in S4 Fig and associated models are shown in Figs 2 and 3. (PDF) [file ppat.1006986.s002.pdf]

**Map**	BG505_crosslinked_PGV04
**EMDB ID**	EMD-7568
**PDB ID**	6CRQ
**Data collection**	
Microscope	FEI Titan Krios
Voltage (kV)	300
Detector	Gatan K2 Summit
Recording mode	Counting
Magnification (incl. post-magnification)	49,020
Movie micrograph pixelsize (Å)	1.02
Dose rate (e^-/[(camera pixel)*s])	10
Number of frames per movie micrograph	35
Frame exposure time (ms)	200
Movie micrograph exposure time (s)	7
Total dose (e^-/Å^2)	66
Defocus range (μm)	1.5-4.0
**EM data processing**	
Number of movie micrographs	1,329
Number of molecular projection images in map	55,563
Symmetry	C3
Map resolution (FSC 0.143; Å)	4.2
Map sharpening B-factor (Å^2)	-166
**Structure Building and Validation**	
Number of atoms in deposited model	20,289
gp41 (peptide; chain C,G,H)	967
gp120 (peptide; chain A,B,F)	3,538
PGV04 VH (chain H)	999
PGV04 VL (chain L)	784
Glycans	475
MolProbity score	1.38 (97%)
Clashscore	5.87
EMRinger score	2.29
Deviations from ideal	
Bond length outliers	0 (0%)
Bond angle outliers	0 (0%)
Monosaccharide outliers	0 (0%)
Ramachandran plot	
Favored (%)	97.7
Allowed (%)	2.3
Outliers (%)	0.0
