# Supplementary figures and images for: Structural and immunologic correlates of chemically stabilized HIV-1 envelope glycoproteins
Source: PLoS Pathog. 2018 May 10;14(5):e1006986. doi: 10.1371/journal.ppat.1006986 (PMC5944921; doi:10.1371/journal.ppat.1006986)

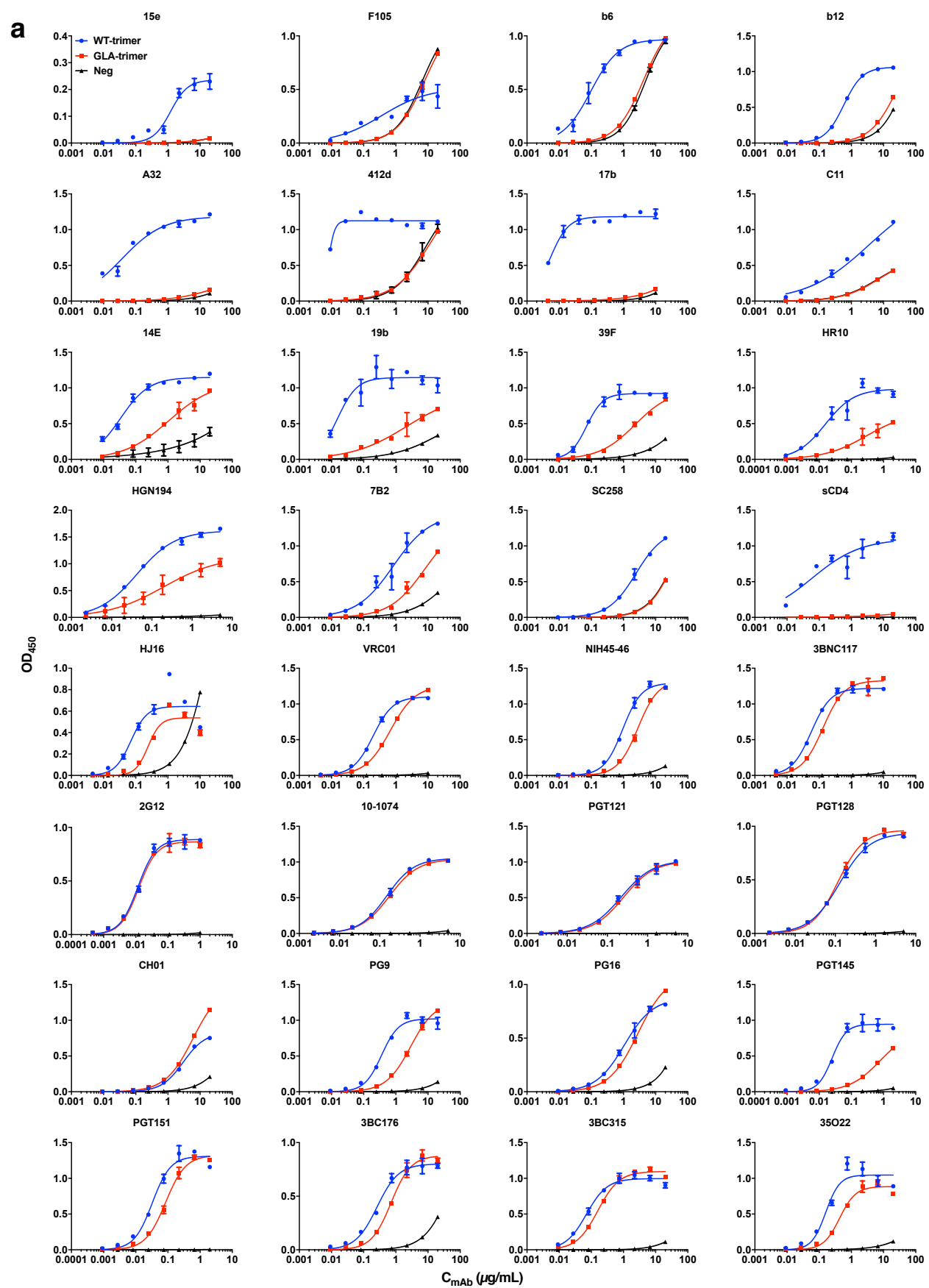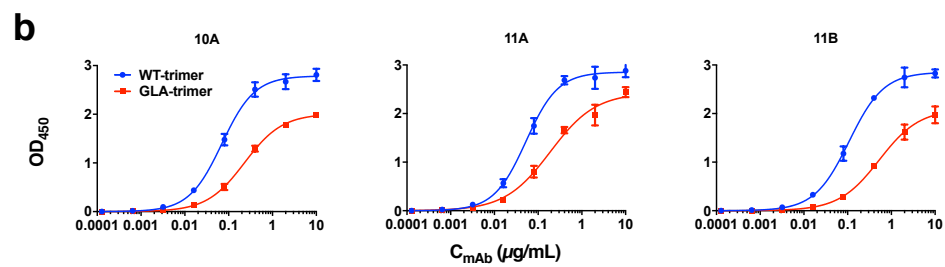

Supplement: S1 Fig — Binding curves of (a) human non-NAbs, bNAbs and sCD4 or (b) rabbit autologous NAbs binding to SOSIP trimer, GLA-SOSIP trimer or BSA (neg) as measured by capture ELISA. ELISA reactions were over-developed for non-NAbs to yield quantifiable binding curves. Curves shown are representative of 2–4 independent experiments. Error bars indicate SD of technical replicates. (PDF) [file ppat.1006986.s003.pdf]

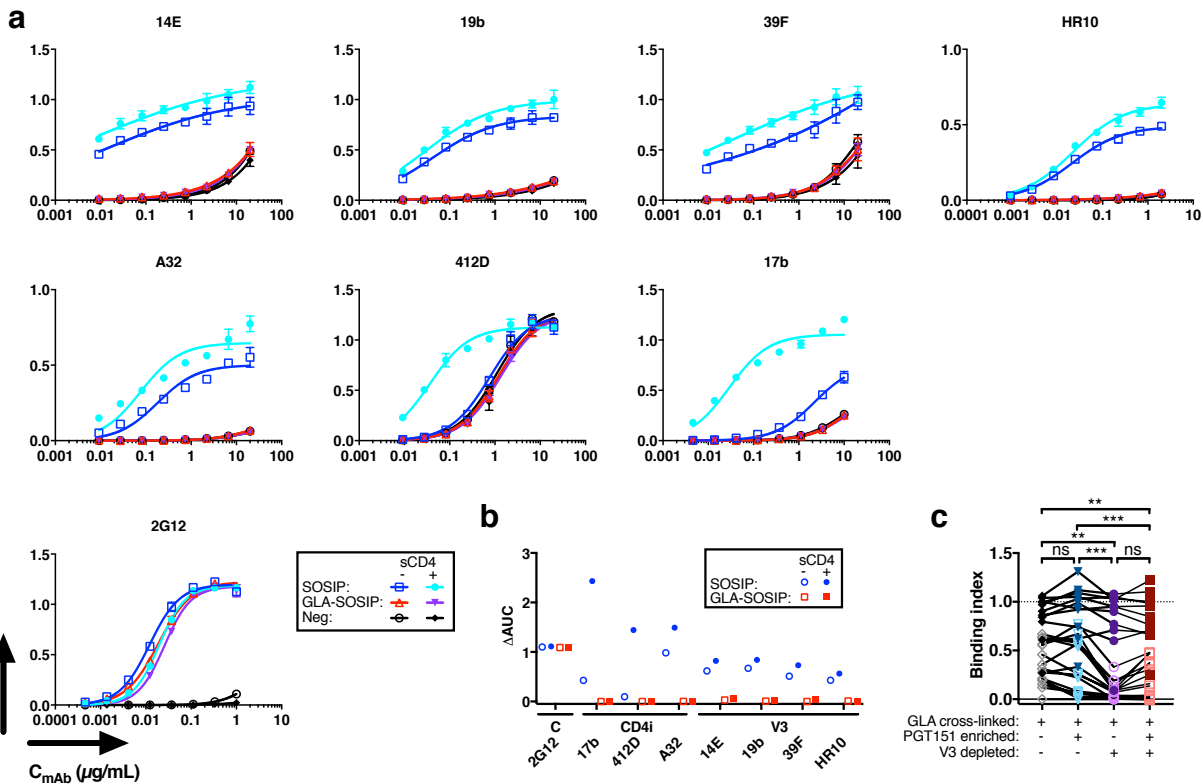

Supplement: S3 Fig — (a) Binding of V3-specific (top row) and sCD4-induced (middle row) non-NAbs to SOSIP and GLA-SOSIP trimer was assayed by capture ELISA. 2G12 (bottom) served as a loading control. Binding curves to SOSIP trimer, GLA-SOSIP trimer or BSA (neg) are shown in presence (solid symbols) and absence (empty symbols) of sCD4. ELISA reactions were over-developed for non-NAbs to yield OD values where possible >1 to allow quantification. Curves shown are representative of 3 independent experiments, error bars indicate SD of technical repeats. (b) sCD4-induced non-Nab binding. Binding of CD4-inducible (CD4i) and V3 non-nAbs to SOSIP-trimers (blue) and GLA-SOSIP-trimers (red) and loading control mAb 2G12 was measured in the presence (filled symbols) or absence (empty symbols) of sCD4 by ELISA. AUC values are defined as background-subtracted area under the curve, data derived from a representative experiment of two independent repeats. (c) Comparison of antigenic profiles of double-selected GLA-SOSIP trimers with previously published [29] unselected and V3-negative or PGT151-positive single-selected cross-linked trimers. bNAbs are shown as dark-colored filled symbols and non-NAbs as light-colored empty symbols. Data are averages of 2–6 independent repeats. **p <0.01, ***p<0.001, ns = not significant, one way ANOVA with Dunn’s multiple correction. (PDF) [file ppat.1006986.s005.pdf]

## BG505-GLA in complex with PGV04

**a**

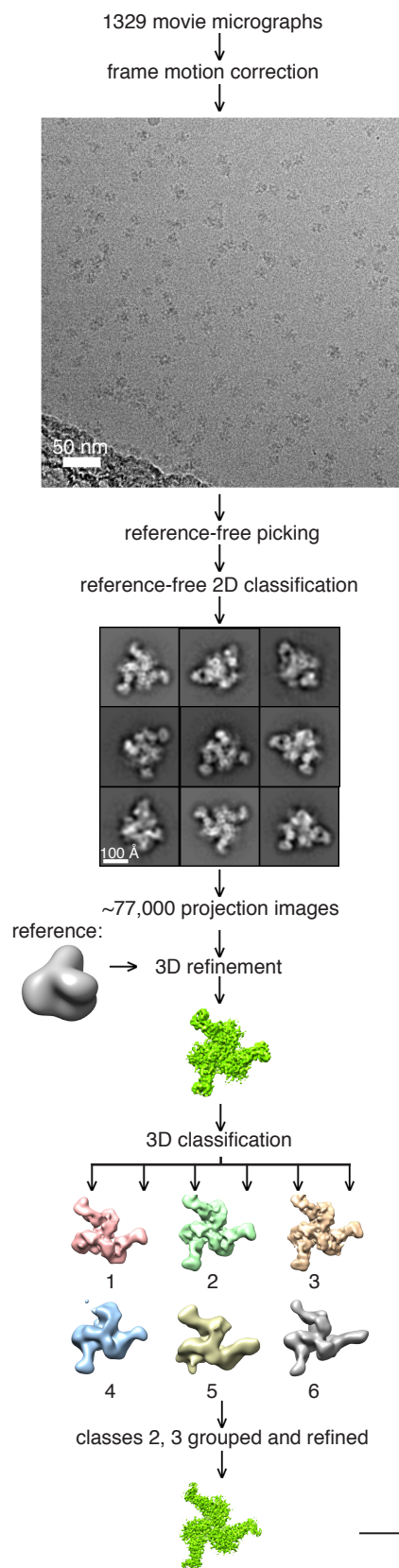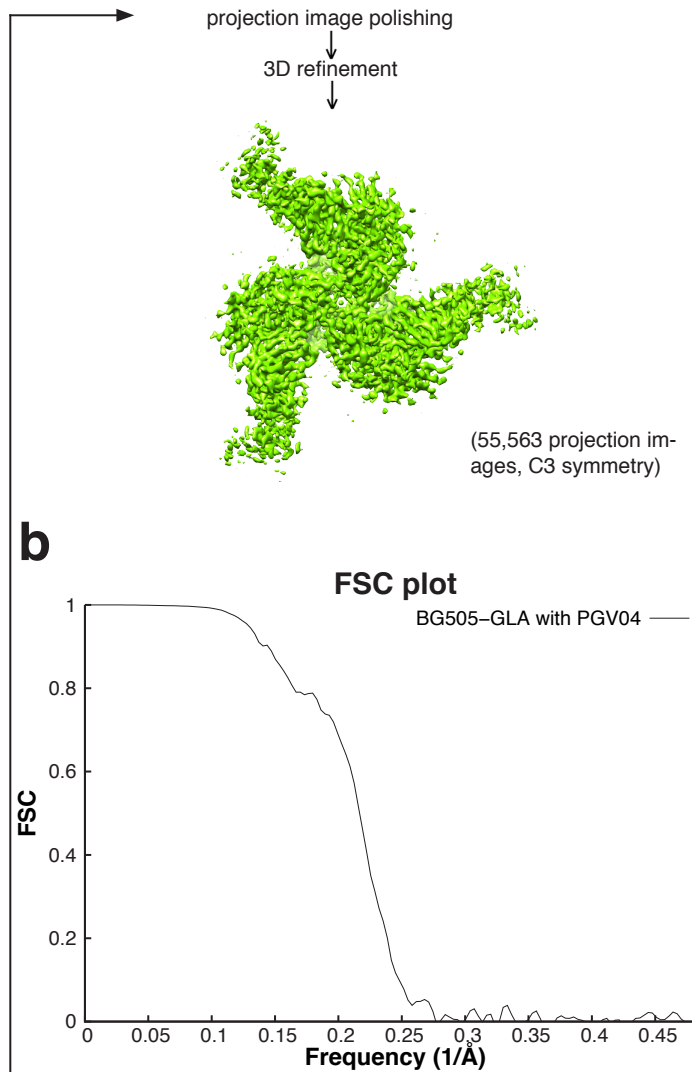

**c**

Relative angular distribution of projection images

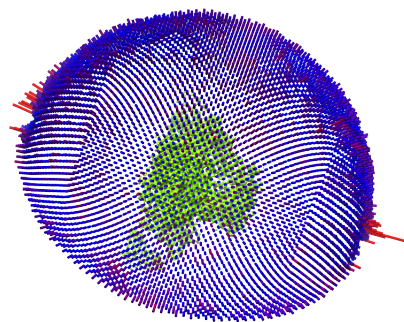

Supplement: S4 Fig — (a) Flow diagram of steps utilized in cryo-EM data processing. The final reconstruction contained 55,563 molecular projection images and was obtained imposing C3 rotational symmetry during refinement. (b) Plot of Fourier shell correlation (FSC) between two independently refined data half sets that were combined into the final reconstructed density map. Globally averaged resolution measured by the 0.143 criterion was 4.2Å. (c) Plot showing angular coverage by the 55,563 molecular projection images that contributed to the final reconstructed density map. (PDF) [file ppat.1006986.s006.pdf]
