# Supplementary material for: Structural and immunologic correlates of chemically stabilized HIV-1 envelope glycoproteins
Source: PLoS Pathog. 2018 May 10;14(5):e1006986. doi: 10.1371/journal.ppat.1006986 (PMC5944921; doi:10.1371/journal.ppat.1006986)
Supplement: S2 Fig — SOSIP-trimer (left) or GLA-SOSIP-trimer (right) were captured onto each flow cell using mAb 2G12 (not shown), and 4-fold dilution series of the indicated Fab starting at 10 μM were passed over the surface followed by buffer only. Vertical lines indicate start and stop of injection. Black curves indicate the fit of a 1:1 binding model, fit values are summarized in S1 Table and results summarized in Fig 1E. (PDF) [file ppat.1006986.s004.pdf]

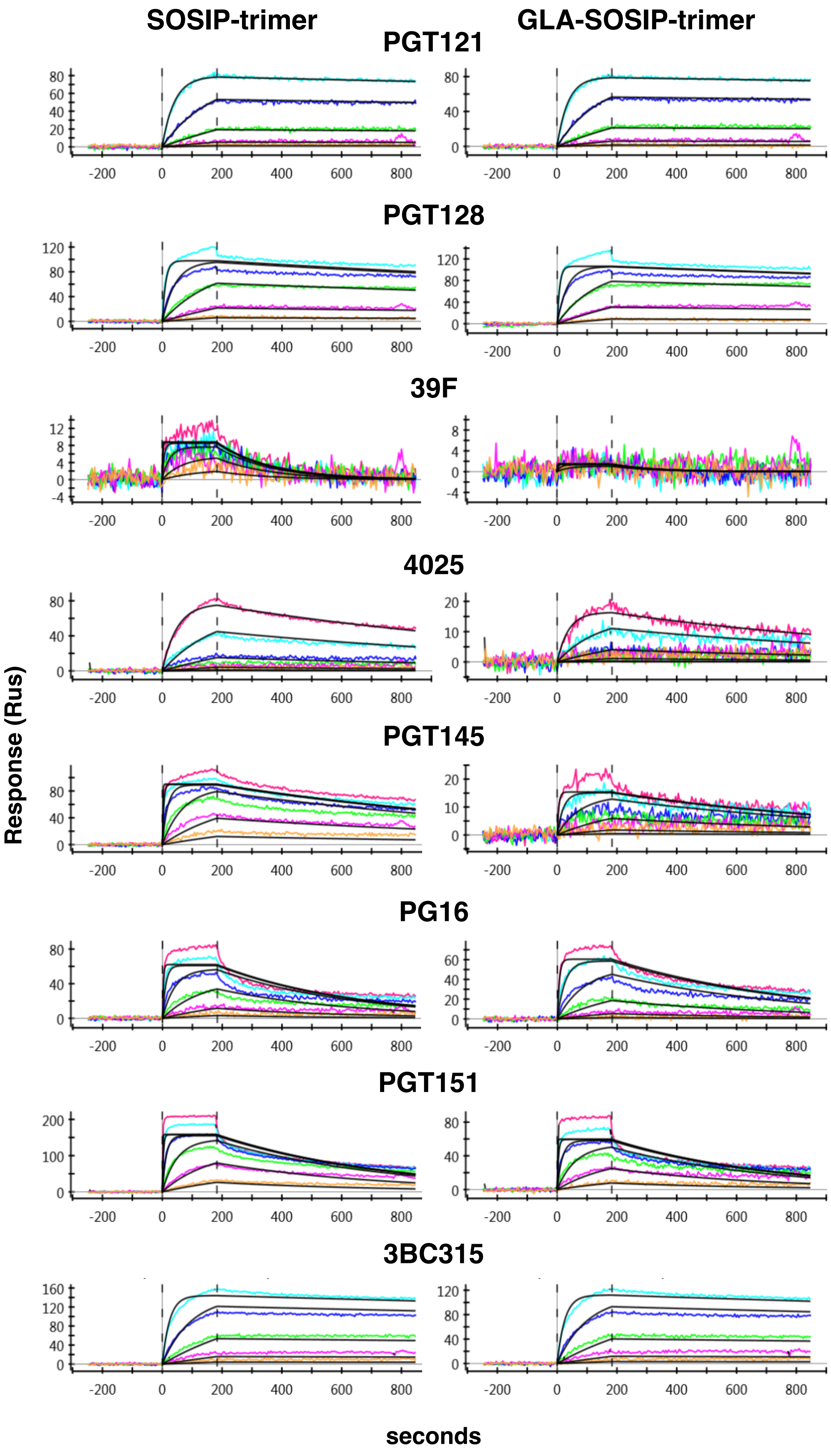

SOSIP-trimer
GLA-SOSIP-trimer
PGT121
PGT128
39F
4025
PGT145
PG16
PGT151
3BC315
Response (Rus)
seconds

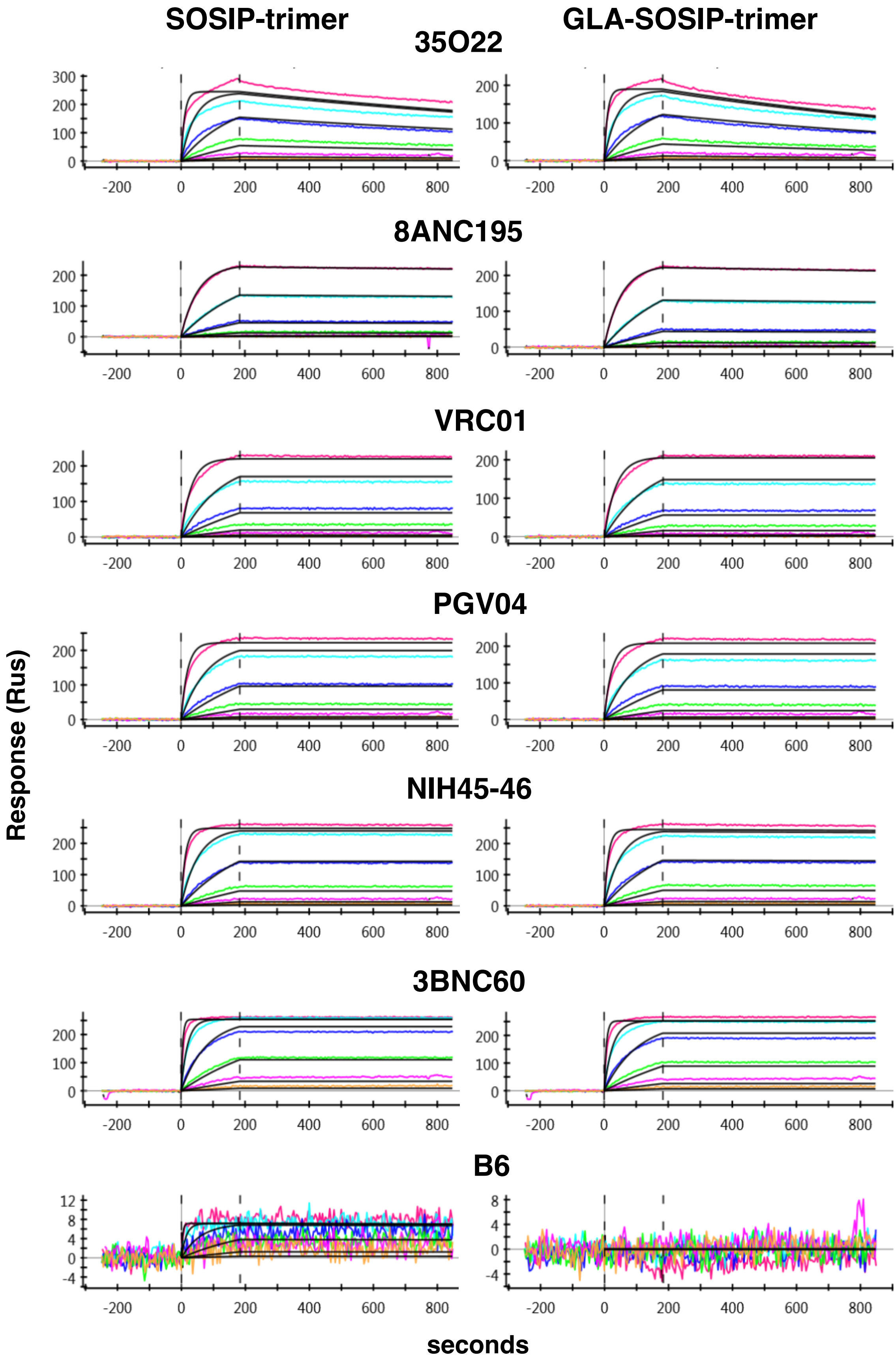
SOSIP-trimer
GLA-SOSIP-trimer
35O22
8ANC195
VRC01
PGV04
NIH45-46
3BNC60
B6
Response (Rus)
seconds
300
200
100
0
-200
400
600
800
12
8
4
-4
