# Supplementary material for: Structural and immunologic correlates of chemically stabilized HIV-1 envelope glycoproteins
Source: PLoS Pathog. 2018 May 10;14(5):e1006986. doi: 10.1371/journal.ppat.1006986 (PMC5944921; doi:10.1371/journal.ppat.1006986)
Supplement: S5 Fig — (a) Serum from WT-trimer-immunized rabbits assayed on gp120 peptide array represented as fluorescent signal intensity. (b) Serum from GLA-trimer-immunized rabbits assayed on gp120 peptide array. (c) Serum from SOSIP trimer-immunized rabbits assayed on gp41 peptide array. (d) Serum from GLA-SOSIP trimer-immunized rabbits assayed on gp41 peptide array. Colored lines represent different clades from which peptides were derived. (PDF) [file ppat.1006986.s007.pdf]

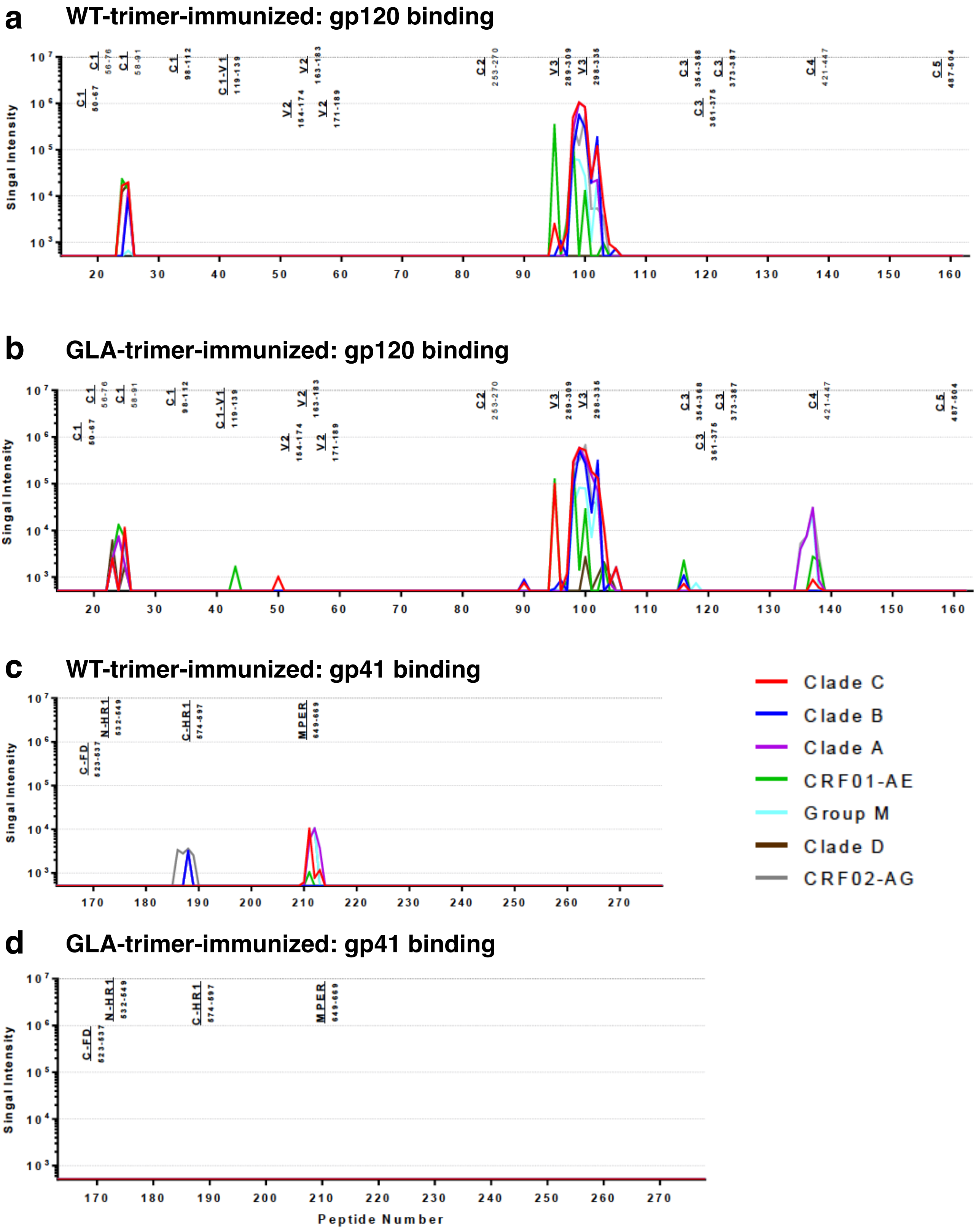
a
WT-trimer-immunized: gp120 binding
Singal Intensity
C1 50-67
C1 56-76
C1 58-91
C1 98-112
C1-V1 119-139
V2 154-174
V2 163-183
V2 171-189
C2 253-270
V3 289-309
V3 298-335
C3 354-368
C3 361-375
C3 373-387
C4 421-447
C5 487-504
b
GLA-trimer-immunized: gp120 binding
Singal Intensity
c
WT-trimer-immunized: gp41 binding
Singal Intensity
C-FD 523-537
N-HR1 532-549
C-HR1 574-597
MPER 649-669
d
GLA-trimer-immunized: gp41 binding
Singal Intensity
Peptide Number
Clade C
Clade B
Clade A
CRF01-AE
Group M
Clade D
CRF02-AG
