# Supplementary material for: Structural and immunologic correlates of chemically stabilized HIV-1 envelope glycoproteins
Source: PLoS Pathog. 2018 May 10;14(5):e1006986. doi: 10.1371/journal.ppat.1006986 (PMC5944921; doi:10.1371/journal.ppat.1006986)
Supplement: S7 Fig — CD4 T cells negatively enriched from BALB/c splenocytes were restimulated in vitro using a 165 peptide library each of 15 amino acids overlapping by 5. Data presented are from two independent experiments. The peptide number is shown alongside the relevant amino acids and the region of gp140 represented. Gold coloring represents the regions in which GLA cross-links were detected in the GLA-SOSIP trimer structure. Proliferation was measured by 3H incorporation, where green = response below baseline, white (1-1000cpm), pink (1001-5000cpm), red (5001-8000cpm). Thick vertical blue bars represent dominant epitope responses (mean >1000) to SOSIP trimer immunization with at least 2 positive adjacent peptide responses in both experiments, thin vertical blue bars represent single peptide positive responses with CPM >1000. Thick vertical red bars represent dominant epitope responses (mean >1000) to GLA-SOSIP trimer immunization, thin vertical red bars represent single peptide positive responses. IFN-γ was assayed by ELISA, where green = response below baseline, white (0.001–1 OD), pale pink (1.001–2 OD), red (2.001–3 OD). IL-4 was assayed by ELISA, where green = response below baseline, white (0.001–0.2), pale pink (0.2001–0.4), red (0.4001–0.6). Criteria for selecting IFN-γ and IL-4 responses were positive responses in both experiments and a mean response across both experiments of >0.1 OD. Vertical blue and red bars represent epitopes eliciting cytokine responses as defined for proliferation. (PDF) [file ppat.1006986.s009.pdf]

Peptide	Amino acids	Region	Proliferation UM Expt 1	UM Expt 2	UM Average	GLA Expt 1	GLA Expt 2	GLA Average
1	(1-15)		0	0	0	0	0	0
2	(5-19)		0	0	0	0	0	0
3	(9-23)		0	0	0	0	0	0
4	(13-27)		0	0	0	0	0	0
5	(17-31)		982	0	491	0	0	0
6	(21-35)		0	0	0	0	0	0
7	(25-39)		0	0	0	0	0	0
8	(29-43)		0	0	0	0	0	0
9	(33-47)		0	0	0	0	24	12
10	(37-51)		0	0	0	0	0	0
11	(41-55)		0	0	0	0	0	0
12	(45-59)		0	3574	1787	0	0	0
13	(49-63)		0	1418	709	0	0	0
14	(53-67)		0	768	384	0	0	0
15	(57-71)		980	282	631	288	0	144
16	(61-75)		0	3870	1935	396	0	198
17	(65-79)	gp120 C1	5121	1883	3502	466	3534	2000
18	(69-83)		5394	1566	3780	982	6018	3500
19	(73-87)		874	0	437	46	464	255
20	(77-91)		0	582	291	0	18	9
21	(81-95)		307	485	396	0	0	0
22	(85-99)		0	0	0	0	0	0
23	(89-103)		0	0	0	0	0	0
24	(93-107)		0	228	114	0	0	0
25	(97-111)		0	1810	905	92	0	46
26	(101-115)		0	1654	827	0	0	0
27	(105-119)		0	712	356	0	0	0
28	(109-123)		29	3003	1516	246	0	123
29	(113-127)		1270	0	635	0	132	66
30	(117-131)		2230	0	1115	0	900	450
31	(121-135)		0	2406	1203	228	0	114
32	(125-139)		0	0	0	0	0	0
33	(129-143)		0	594	297	0	0	0
34	(133-147)		2632	0	1316	0	0	0
35	(137-151)		0	574	287	0	186	93
36	(141-155)		0	454	227	0	0	0
37	(145-159)	V1	0	752	376	0	0	0
38	(149-163)		158	0	79	0	0	0
39	(153-167)		0	0	0	0	0	0
40	(157-171)		629	341	485	0	0	0
41	(161-175)		0	0	0	0	0	0
42	(165-179)		114	0	57	0	0	0
43	(169-183)		0	0	0	0	0	0
44	(173-187)		0	0	0	0	0	0
45	(177-191)	V2	276	864	570	0	0	0
46	(181-195)		628	340	484	0	0	0
47	(185-199)		0	0	0	834	0	417
48	(189-203)		0	2532	1266	1898	0	949
49	(193-207)		3286	0	1643	0	266	133
50	(197-211)		0	2280	1140	0	0	0
51	(201-215)		0	1364	682	0	0	0
52	(205-219)		0	4620	2310	528	0	264
53	(209-223)		1019	7481	4250	3666	0	1833
54	(213-227)		3293	2455	2874	1462	1160	1311
55	(217-231)		2056	0	1028	0	12	6
56	(221-235)		0	0	0	0	0	0
57	(225-239)		269	95	182	0	0	0
58	(229-243)		0	876	438	0	0	0
59	(233-247)		0	738	369	0	204	102
60	(237-251)		0	0	0	0	0	0
61	(241-255)		2533	805	1669	0	896	448
62	(245-259)	C2	2	4234	2118	466	3934	2200
63	(249-263)		231	1543	887	0	286	143
64	(253-267)		1007	427	717	0	0	0
65	(257-271)		2828	0	1414	12	0	6
66	(261-275)		1930	0	965	0	0	0
67	(265-279)		0	1836	918	0	0	0
68	(269-283)		994	1288	1141	0	0	0
69	(273-287)		1437	899	1168	0	0	0
70	(277-291)		0	490	245	0	0	0
71	(281-295)		0	0	0	0	0	0
72	(285-299)		0	0	0	0	0	0
73	(289-303)		149	1093	621	0	216	108
74	(293-307)		288	852	570	0	0	0
75	(297-311)		0	0	0	0	0	0
76	(301-315)		0	158	79	0	0	0
77	(305-319)		0	852	426	0	0	0
78	(309-323)	V3	0	3132	1566	0	0	0
79	(313-327)		1240	0	620	0	0	0
80	(317-331)		2506	0	1253	0	0	0
81	(321-335)		572	1494	1033	0	0	0
82	(325-339)		0	0	0	0	0	0
83	(329-343)		0	938	469	0	0	0
84	(333-347)		0	0	0	0	0	0
85	(337-351)		0	0	0	0	0	0
86	(341-355)		0	0	0	0	0	0
87	(345-359)		0	0	0	0	0	0
88	(349-363)		0	0	0	0	0	0
89	(353-367)		0	840	420	0	0	0
90	(357-371)	C3	424	1506	965	0	0	0
91	(361-375)		0	0	0	0	0	0
92	(365-379)		508	1826	1167	0	0	0
93	(369-383)		0	656	328	0	0	0
94	(373-387)		0	0	0	0	594	297
95	(377-391)		0	1024	512	0	1260	630
96	(381-395)		0	0	0	0	0	0
97	(385-399)		0	0	0	0	0	0
98	(389-403)		0	0	0	0	0	0
99	(393-407)		0	0	0	0	0	0
100	(397-411)		0	180	90	0	0	0
101	(401-415)	V4	0	0	0	0	0	0
102	(405-419)		852	6274	3563	338	2462	1400
103	(409-423)		0	0	0	0	0	0
104	(413-427)		0	0	0	0	0	0
105	(417-431)		0	0	0	0	0	0
106	(421-435)		0	0	0	0	0	0
107	(425-439)		0	0	0	0	0	0
108	(429-443)		0	0	0	0	0	0
109	(433-447)		0	962	481	0	0	0
110	(437-451)	C4	0	0	0	0	48	24
111	(441-455)		0	0	0	0	0	0
112	(445-459)		0	0	0	0	0	0
113	(449-463)		0	0	0	0	0	0
114	(453-467)		0	1076	538	2128	0	1064
115	(457-471)		0	0	0	0	0	0
116	(461-475)		0	0	0	85	313	199
117	(465-479)	V5	376	0	188	0	0	0
118	(469-483)		0	0	0	0	0	0
119	(473-487)		0	0	0	0	0	0
120	(477-491)		0	0	0	0	0	0
121	(481-495)		408	2092	1250	0	806	403
122	(485-499)		0	2896	1448	0	0	0
123	(489-503)	C5	0	0	0	60	0	30
124	(493-507)		0	0	0	0	0	0
125	(497-511)		0	2294	1147	1490	0	745
126	(501-515)		644	4218	2431	0	0	0
127	(505-519)		0	2106	1053	0	0	0
128	(509-523)		0	0	0	0	0	0
129	(513-527)		1192	0	596	0	216	108
130	(517-531)		0	1240	620	0	0	0
131	(521-535)		1002	0	501	0	0	0
132	(525-539)		1239	27	633	0	0	0
133	(529-543)		0	0	0	0	0	0
134	(533-547)		0	0	0	0	0	0
135	(537-551)		0	0	0	0	0	0
136	(541-555)		0	0	0	0	0	0
137	(545-559)		98	0	49	0	0	0
138	(549-563)		0	0	0	0	0	0
139	(553-567)		250	536	393	0	0	0
140	(557-571)		0	0	0	0	0	0
141	(561-575)		750	1010	880	0	26	13
142	(565-579)		0	516	258	0	0	0
143	(569-583)		182	0	91	0	0	0
144	(573-587)		0	0	0	0	1946	973
145	(577-591)		2424	0	1212	0	696	348
146	(581-595)		1622	0	811	98	0	49
147	(585-599)	gp41	0	0	0	1314	0	657
148	(589-603)		0	0	0	0	0	0
149	(593-607)		278	0	139	0	0	0
150	(597-611)		0	680	340	0	0	0
151	(601-615)		0	848	424	0	672	336
152	(605-619)		1983	1175	1579	0	2200	1100
153	(609-623)		0	3916	1958	860	0	430
154	(613-627)		0	194	97	0	0	0
155	(617-631)		0	0	0	0	0	0
156	(621-635)		0	0	0	0	0	0
157	(625-639)		0	0	0	0	0	0
158	(629-643)		0	0	0	0	0	0
159	(633-647)		0	0	0	0	0	0
160	(637-651)		0	0	0	0	0	0
161	(641-655)		213	1547	880	0	0	0
162	(645-659)		0	62	31	92	0	46
163	(649-663)		1472	164	818	0	0	0
164	(653-667)		0	1336	668	0	0	0
165	(657-671)		0	480	240	0	0	0

Peptide	IFN-γ UM Expt 1	UM Expt 2	UM Average	GLA Expt 1	GLA Expt 2	GLA Average
1	0	0	0	0	0	0
2	0	0	0	0	0	0
3	0	0	0	0	0	0
4	0	0	0	0	0	0
5	0	0	0	0	0	0
6	0.09	0.17	0.13	0	0	0
7	0	0	0	0	0.184	0.092
8	0	0	0	0.023	0.023	0.023
9	0	0	0	0	0	0
10	0	0	0	0.05	0.122	0.086
11	0	0	0	0.057	0.057	0.057
12	0	0.504	0.252	0	0	0
13	0	0	0	0	0	0
14	0	0	0	0	0	0
15	0	0	0	0.056	0.216	0.136
16	0.05	0.298	0.174	0	0.02	0.01
17	0.068	0.336	0.202	0.066	0.066	0.066
18	0.035	0.136	0.0855	0.118	0.118	0.118
19	0.124	0.54	0.332	0.038	0.204	0.121
20	0.039	0.372	0.2055	0.051	0.051	0.051
21	0.08	0.38	0.23	0.08	0.216	0.148
22	0	0	0	0	0	0
23	0	0	0	0	0	0
24	0	0	0	0	0.04	0.02
25	0	0	0	0	0.084	0.042
26	0.036	0.476	0.256	0	0	0
27	0	0.232	0.116	0.105	0.105	0.105
28	0	0.444	0.222	0.032	0.032	0.032
29	0	0.346	0.173	0.042	0.322	0.182
30	0.06	0.406	0.233	0.122	0.348	0.235
31	0.09	0.09	0.09	0.04	0.04	0.04
32	0	0	0	0	0	0
33	0.051	0.051	0.051	0.039	0.039	0.039
34	0	0.344	0.172	0	0	0
35	0	0	0	0	0	0
36	0	0	0	0	0	0
37	0	0	0	0	0	0
38	0	0	0	0.094	0.242	0.168
39	0	0	0	0	0	0
40	0	0.924	0.462	0.058	0.08	0.069
41	0.106	0.282	0.194	0	0	0
42	0	0	0	0	0	0
43	0.08	0.246	0.163	0.056	0.056	0.056
44	0	0	0	0	0	0
45	0	0.112	0.056	0	0.072	0.036
46	0	0	0	0	0.136	0.068
47	0	0	0	0	0.042	0.021
48	0	0.114	0.057	0	0.154	0.077
49	0	0	0	0.059	0.059	0.059
50	0.148	0.932	0.54	0.112	0.254	0.183
51	0.232	0.456	0.344	0.039	0.039	0.039
52	0.154	2.256	1.205	0.24	2.078	1.159
53	0.612	1.666	1.139	0.784	2.876	1.83
54	0.716	1.552	1.134	0.14	0.14	0.14
55	0.508	0	0.254	0.055	0.055	0.055
56	0	0.528	0.264	0	0.054	0.027
57	0.138	0.212	0.175	0	0	0
58	0	0	0	0	0.08	0.04
59	0	0	0	0	0	0
60	0	0	0	0	0	0
61	0.084	0.086	0.085	0	0	0
62	0.158	0.37	0.264	0.078	0.078	0.078
63	0.315	0.315	0.315	0.06	0.06	0.06
64	0.2	0.2	0.2	0.028	0.028	0.028
65	0.132	0.296	0.214	0.022	0.11	0.066
66	0.2	1.894	1.047	0.03	0.03	0.03
67	0	0.216	0.108	0.02	0.114	0.067
68	0	0.39	0.195	0.034	0.144	0.089
69	0	0	0	0	0	0
70	0	0	0	0	0	0
71	0	0	0	0	0	0
72	0	0	0	0	0	0
73	0.077	0.077	0.077	0	0	0
74	0.067	0.067	0.067	0	0	0
75	0.093	0.093	0.093	0.062	0.084	0.073
76	0.11	0.316	0.213	0.082	0.082	0.082
77	0.005	0.005	0.005	0.055	0.055	0.055
78	0	0.47	0.235	0	0	0
79	0	0.33	0.165	0	0	0
80	0	0.2	0.1	0	0	0
81	0	0.352	0.176	0	0	0
82	0	0.278	0.139	0	0	0
83	0	0	0	0	0.034	0.017
84	0	0	0	0	0	0
85	0	0	0	0.019	0.019	0.019
86	0	0	0	0	0	0
87	0	0	0	0	0	0
88	0	0	0	0	0	0
89	0	0.222	0.111	0	0.11	0.055
90	0.142	0.186	0.164	0	0	0
91	0	0	0	0	0	0
92	0	0	0	0	0	0
93	0	0	0	0	0	0
94	0	0	0	0	0	0
95	0	0	0	0	0.024	0.012
96	0	0	0	0	0	0
97	0	0	0	0	0	0
98	0	0	0	0	0	0
99	0	0	0	0	0	0
100	0	0	0	0	0.028	0.014
101	0	0	0	0.086	0.086	0.086
102	0	0	0	0	0	0
103	0	0	0	0.572	1.758	1.165
104	0	0	0	0	0	0
105	0	0	0	0	0.282	0.141
106	0	0	0	0	0	0
107	0	0	0	0	0	0
108	0	0	0	0	0	0
109	0	0	0	0	0	0
110	0	0	0	0.034	0.034	0.034
111	0	0.054	0.027	0	0	0
112	0	0.308	0.154	0	0.27	0.135
113	0	0	0	0	0	0
114	0	0.118	0.059	0.074	0.074	0.074
115	0	0	0	0.026	0.026	0.026
116	0	0	0	0.094	0.144	0.119
117	0	0	0	0.053	0.053	0.053
118	0	0	0	0	0	0
119	0	0	0	0.066	0.066	0.066
120	0	0	0	0.083	0.083	0.083
121	0	0	0	0.08	0.08	0.08
122	0.306	0.306	0.306	0.189	0.189	0.189
123	0.121	0.121	0.121	0.152	0.152	0.152
124	0	0	0	0	0	0
125	0	0.152	0.076	0.008	0.066	0.037
126	0	0.262	0.131	0	0	0
127	0	0.356	0.178	0.034	0.282	0.158
128	0	0	0	0	0	0
129	0	0.124	0.062	0	0.13	0.065
130	0	0	0	0	0	0
131	0	0	0	0.045	0.045	0.045
132	0	0	0	0.097	0.097	0.097
133	0	0	0	0.047	0.047	0.047
134	0.13	0.13	0.13	0.01	0.01	0.01
135	0	0	0	0	0	0
136	0	0.138	0.069	0	0	0
137	0	0	0	0	0	0
138	0	0	0	0.085	0.085	0.085
139	0	0.146	0.073	0.091	0.091	0.091
140	0	0	0	0.056	0.18	0.118
141	0	0.406	0.203	0.07	0.07	0.07
142	0.138	0.138	0.138	0.098	0.098	0.098
143	0	0	0	0.008	0.008	0.008
144	0	0	0	0.103	0.103	0.103
145	0	0	0	0.041	0.041	0.041
146	0.191	0.191	0.191	0.151	0.151	0.151
147	0.087	0.087	0.087	0.032	0.526	0.279
148	0.066	0.066	0.066	0.049	0.049	0.049
149	0	0.404	0.202	0.038	0.038	0.038
150	0.05	0.326	0.188	0.146	0.146	0.146
151	0.202	0.202	0.202	0.038	0.342	0.19
152	0	0	0	0.008	0.526	0.267
153	0.02	0.284	0.152	0	0	0
154	0	0	0	0	0.136	0.068
155	0	0	0	0.007	0.007	0.007
156	0	0	0	0	0	0
157	0	0	0	0	0	0
158	0	0	0	0	0.124	0.062
159	0	0	0	0.084	0.084	0.084
160	0	0	0	0	0.042	0.021
161	0	0.166	0.083	0.088	0.088	0.088
162	0.012	0.284	0.148	0.066	0.242	0.154
163	0	0.898	0.449	0.054	0.174	0.114
164	0	0.216	0.108	0.029	0.029	0.029
165	0	0	0	0	0	0

Peptide	IL-4 UM Expt 1	UM Expt 2	UM Average	GLA Expt 1	GLA Expt 2	GLA Average
1	0	0	0	0.016	0.024	0.02
2	0	0	0	0	0	0
3	0	0	0	0.027	0.031	0.029
4	0	0	0	0.067	0.071	0.069
5	0	0	0	0.087	0.097	0.092
6	0.036	0.054	0.045	0.028	0.046	0.037
7	0	0.092	0.046	0	0	0
8	0	0	0	0.046	0.104	0.075
9	0	0	0	0.02	0.024	0.022
10	0	0	0	0.015	0.035	0.025
11	0	0	0	0.04	0.064	0.052
12	0	0	0	0.018	0.016	0.017
13	0	0	0	0	0	0
14	0	0	0	0.021	0.039	0.03
15	0	0	0	0.002	0.054	0.028
16	0	0	0	0	0	0
17	0.006	0.062	0.034	0.06	0.144	0.102
18	0	0	0	0.096	0.126	0.111
19	0.009	0.115	0.062	0	0.038	0.019
20	0	0	0	0.03	0.036	0.033
21	0	0	0	0.03	0.05	0.04
22	0	0	0	0	0	0
23	0	0	0	0	0	0
24	0	0	0	0	0	0
25	0.007	0.035	0.021	0	0	0
26	0.01	0.026	0.018	0.016	0.058	0.037
27	0	0	0	0.055	0.095	0.075
28	0	0	0	0.015	0.023	0.019
29	0	0	0	0	0.042	0.021
30	0.01	0.05	0.03	0.042	0.08	0.061
31	0.051	0.161	0.106	0.041	0.125	0.083
32	0	0	0	0	0.04	0.02
33	0.012	0.092	0.052	0.038	0.118	0.078
34	0	0	0	0	0	0
35	0	0	0	0	0	0
36	0	0	0	0.007	0.017	0.012
37	0	0	0	0.005	0.009	0.007
38	0	0	0	0.043	0.051	0.047
39	0	0	0	0.054	0.062	0.058
40	0	0	0	0.023	0.035	0.029
41	0.032	0.074	0.053	0.028	0.038	0.033
42	0	0	0	0	0	0
43	0.03	0.05	0.04	0.025	0.033	0.029
44	0	0	0	0.012	0.016	0.014
45	0	0	0	0	0	0
46	0	0	0	0	0	0
47	0	0	0	0	0	0
48	0	0	0	0	0	0
49	0.22	0.244	0.232	0.097	0.121	0.109
50	0.063	0.085	0.074	0.035	0.077	0.056
51	0.036	0.196	0.116	0.03	0.046	0.038
52	0.032	0.122	0.077	0.048	0.072	0.06
53	0.25	0.362	0.306	0.117	0.275	0.196
54	0.355	0.361	0.358	0.241	0.249	0.245
55	0.197	0.327	0.262	0.072	0.112	0.092
56	0	0	0	0	0	0
57	0.044	0.094	0.069	0.039	0.047	0.043
58	0	0	0	0	0	0
59	0	0	0	0	0	0
60	0	0	0	0	0	0
61	0.018	0.066	0.042	0	0	0
62	0.014	0.144	0.079	0.052	0.076	0.064
63	0.075	0.325	0.2	0.123	0.219	0.171
64	0.167	0.175	0.171	0.053	0.085	0.069
65	0.041	0.091	0.066	0.009	0.013	0.011
66	0.037	0.163	0.1	0.148	0.154	0.151
67	0	0	0	0	0.02	0.01
68	0	0	0	0.013	0.021	0.017
69	0	0	0	0	0	0
70	0	0	0	0	0	0
71	0	0	0	0	0	0
72	0.099	0.249	0.174	0.054	0.08	0.067
73	0.113	0.137	0.125	0.027	0.043	0.035
74	0.037	0.089	0.063	0.017	0.081	0.049
75	0.075	0.131	0.103	0.023	0.039	0.031
76	0.049	0.061	0.055	0.035	0.047	0.041
77	0.026	0.086	0.056	0.041	0.059	0.05
78	0	0	0	0	0	0
79	0	0	0	0	0.004	0.002
80	0	0	0	0	0	0
81	0	0	0	0	0	0
82	0	0	0	0	0	0
83	0	0	0	0	0	0
84	0	0	0	0.003	0.007	0.005
85	0	0	0	0.015	0.117	0.066
86	0	0	0	0.023	0.061	0.042
87	0	0	0	0.04	0.038	0.039
88	0	0	0	0.018	0.024	0.021
89	0	0	0	0	0	0
90	0.07	0.072	0.071	0.16	0.244	0.202
91	0	0	0	0	0	0
92	0	0	0	0	0	0
93	0	0	0	0.044	0.046	0.045
94	0.025	0.039	0.032	0	0	0
95	0	0	0	0	0	0
96	0	0	0	0	0	0
97	0	0	0	0.034	0.042	0.038
98	0	0	0	0.016	0.042	0.029
99	0	0	0	0.04	0.062	0.051
100	0	0	0	0	0	0
101	0	0	0	0.083	0.183	0.133
102	0	0	0	0.04	0.074	0.057
103	0.07	0.072	0.071	0.142	0.144	0.143
104	0.021	0.023	0.022	0	0.002	0.001
105	0.072	0.264	0.168	0	0	0
106	0.157	0.437	0.297	0.15	0.19	0.17
107	0	0	0	0	0	0
108	0	0	0	0.015	0.015	0.015
109	0	0	0	0.008	0.076	0.042
110	0.004	0.02	0.012	0.012	0.074	0.043
111	0	0	0	0.033	0.101	0.067
112	0	0	0	0	0	0
113	0.022	0.086	0.054	0.015	0.045	0.03
114	0	0	0	0.029	0.059	0.044
115	0	0	0	0.023	0.049	0.036
116	0	0	0	0.046	0.048	0.047
117	0	0	0	0.044	0.032	0.038
118	0	0	0	0.013	0.029	0.021
119	0	0	0	0.057	0.059	0.058
120	0.012	0.028	0.02	0.044	0.042	0.043
121	0.013	0.017	0.015	0.033	0.061	0.047
122	0.282	0.508	0.395	0.122	0.132	0.127
123	0.221	0.353	0.287	0.177	0.181	0.179
124	0	0	0	0	0	0
125	0	0	0	0	0.008	0.004
126	0	0	0	0.049	0.049	0.049
127	0	0	0	0	0.034	0.017
128	0	0	0	0.037	0.049	0.043
129	0	0	0	0	0	0
130	0.122	0.156	0.139	0.005	0.065	0.035
131	0.042	0.142	0.092	0.046	0.072	0.059
132	0.061	0.101	0.081	0.038	0.066	0.052
133	0.093	0.105	0.099	0.042	0.054	0.048
134	0.075	0.091	0.083	0.055	0.069	0.062
135	0.025	0.031	0.028	0.028	0.038	0.033
136	0	0	0	0	0	0
137	0.04	0.08	0.06	0	0	0
138	0.067	0.089	0.078	0.051	0.073	0.062
139	0	0	0	0.026	0.102	0.064
140	0	0	0	0.018	0.038	0.028
141	0	0	0	0.039	0.087	0.063
142	0.227	0.265	0.246	0.085	0.127	0.106
143	0.174	0.29	0.232	0.124	0.142	0.133
144	0.227	0.285	0.256	0.083	0.165	0.124
145	0.248	0.264	0.256	0.113	0.149	0.131
146	0.136	0.204	0.17	0.076	0.084	0.08
147	0.124	0.178	0.151	0.008	0.024	0.016
148	0.08	0.16	0.12	0.024	0.04	0.032
149	0	0	0	0.02	0.08	0.05
150	0	0.05	0.025	0.066	0.124	0.095
151	0.122	0.142	0.132	0.016	0.022	0.019
152	0	0	0	0	0.008	0.004
153	0.004	0.016	0.01	0.02	0.034	0.027
154	0	0	0	0	0	0
155	0.115	0.127	0.121	0.02	0.018	0.019
156	0.065	0.105	0.085	0.122	0.146	0.134
157	0	0	0	0.018	0.036	0.027
158	0	0	0	0	0	0
159	0.093	0.155	0.124	0.088	0.182	0.135
160	0.042	0.116	0.079	0	0	0
161	0	0	0	0.044	0.056	0.05
162	0	0.012	0.006	0	0.066	0.033
163	0	0	0	0.023	0.031	0.027
164	0	0	0	0	0.032	0.016
165	0	0	0	0	0.032	0.016

Key:

Proliferation	IFN-γ	IL-4
0	0	0
1-1000	0.001-1	0.001-0.2
1001-5000	1.001-2	0.2001-0.4
5001-8000	2.001-3	0.4001-0.6
