# Supplementary material for: Structural and immunologic correlates of chemically stabilized HIV-1 envelope glycoproteins
Source: PLoS Pathog. 2018 May 10;14(5):e1006986. doi: 10.1371/journal.ppat.1006986 (PMC5944921; doi:10.1371/journal.ppat.1006986)
Supplement: S8 Fig — Data summarized from S7 Fig. Grey shading represents the 5 major epitope clusters (labeled 1–5) observed for Th cell proliferation and/or IFN-γ and/or IL-4 production. (PDF) [file ppat.1006986.s010.pdf]

				Proliferation		IFNγ		IL-4	
**Epitope cluster**	**Peptides**	**Epitope (aa)**	**Epitope region**	**SOSIP**	**GLA**	**SOSIP**	**GLA**	**SOSIP**	**GLA**
1	16-17	61-79	gp120 C1						
	17-18	65-83	gp120 C1						
	18-19	69-87	gp120 C1						
	19-21	73-95	gp120 C1						
	29-30	113-131	gp120 C1						
2	50-54	197-227	gp120 C2						
	52-54	205-227	gp120 C2						
	53-54	209-227	gp120 C2						
	53-55	209-231	gp120 C2						
3	61-62	241-259	gp120 C2						
	62-66	245-271	gp120 C2						
	63-64	249-267	gp120 C2						
	68-69	269-287	gp120 C2						
	72-73	285-303	gp120 C2						
	105-106	417-435	gp120 C4/V4						
	122-123	485-503	gp120 C5						
4	142-145	565-591	gp41 HR1						
	142-148	565-603	gp41 HR1						
	146-147	581-599	gp41 HR1						
5	150-151	597-615	gp41 HR1						
	150-152	597-619	gp41 HR1						
	162-163	641-669	gp41 HR2						

No response

Positive response

Lost response

De-novo response
